# Guidelines for treatment of renal injury during cancer chemotherapy 2016

**DOI:** 10.1007/s10157-017-1448-z

**Published:** 2017-08-30

**Authors:** Shigeo Horie, Mototsugu Oya, Masaomi Nangaku, Yoshinari Yasuda, Yasuhiro Komatsu, Motoko Yanagita, Yuko Kitagawa, Hiroyuki Kuwano, Hiroyuki Nishiyama, Chikashi Ishioka, Hiromasa Takaishi, Hideki Shimodaira, Akira Mogi, Yuichi Ando, Koji Matsumoto, Daisuke Kadowaki, Satoru Muto

**Affiliations:** 1 Department of Urology, Juntendo University Graduate School of Medicine, 2-1-1, Hongo, Bunkyo-ku, Tokyo, 113-8421 Japan; 2 Department of Advanced Informatics for Genetic Disease, Juntendo University Graduate School of Medicine, Tokyo, Japan; 3 Department of Urology, Keio University School of Medicine, Tokyo, Japan; 4 Division of Nephrology and Endocrinology, The University of Tokyo Graduate School of Medicine, Tokyo, Japan; 5 Department of CKD Initiatives/Nephrology, Nagoya University Graduate School of Medicine, Nagoya, Japan; 6 Division of Nephrology, Department of Medicine, St. Luke’s International Hospital, Tokyo, Japan; 7 Department of Nephrology, Kyoto University Graduate School of Medicine, Kyoto, Japan; 8 Department of Surgery, Keio University School of Medicine, Tokyo, Japan; 9 Department of General Surgical Science, Gunma University Graduate School of Medicine, Gunma, Japan; 10 Department of Urology, Faculty of Medicine, University of Tsukuba, Ibaraki, Japan; 11 Department of Clinical Oncology, Institute of Development, Aging and Cancer, Tohoku University, Miyagi, Japan; 12 Keio Cancer Center, Keio University School of Medicine, Tokyo, Japan; 13 Department of Clinical Oncology, Institute of Development, Aging and Cancer, Tohoku University, Miyagi, Japan; 14 Department of Clinical Oncology and Chemotherapy, Nagoya University Hospital, Aichi, Japan; 15 Division of Medical Oncology, Hyogo Cancer Center, Hyogo, Japan; 16 Department of Clinical Pharmacology, Faculty of Pharmaceutical Sciences, Kumamoto University, Kumamoto, Japan

## 1. Introduction

Advances in cancer drug therapy have led to improvements in the outcomes of cancer patients, as well as increasing numbers of patients undergoing anticancer chemotherapy and molecularly targeted drug therapy. One adverse event associated with cancer drug therapy is nephrotoxicity, which impedes effective cancer therapy and diminishes the quality of life of cancer patients. Consequently, onco-nephrology has emerged as a new clinical field concerned with the management of nephrotoxicity in cancer drug therapy, creating expectations for advanced expertise and the accumulation of accurate evidence. However, while patients with renal impairment have heretofore undergone planning regarding administration of cancer drug therapy, procedures for nephropathy prevention, and measures for treatment of drug-induced nephrotoxicity in clinical settings based on tradition, experimental rules, and information from clinical trials, the soundness of the evidence for these practices has been uncertain.

Over the past 10 years, estimated glomerular filtration rate (eGFR) has replaced creatinine clearance in the assessment of renal function; in addition, research has revealed the pathologies of and risk factors for chronic kidney disease (CKD) and acute kidney injury (AKI). The objectives of the guidelines presented here are to support improvements in the results of cancer drug therapy and the quality of life of cancer patients through application of these advances in clinical nephrology and the practice of evidence-based treatment.

For these guidelines, we have assembled a group of Japanese experts on cancer drug therapy and nephrology to select highly important clinical questions that are frequently encountered in everyday practice. These guidelines ultimately comprise 16 clinical questions in two chapters regarding assessment of renal function and prevention of nephropathy during cancer drug therapy, thereby determining the level of evidence to support clinical assessments and elucidating the nature of current standard treatments. However, in drafting these guidelines, we discovered a number of clinical issues (evidence gaps) regarding cancer drug therapy and renal impairment. For example, 1) there is very little clinical research on cancer drug therapy and nephropathy to begin with; 2) many clinical trials continue to use creatinine clearance to assess renal function; 3) in assessments of renal function in large populations, there is a vast discrepancy between eGFR and measured values of GFR; and 4) it remains unknown whether body surface area corrections of drug doses are appropriate for elderly patients (who have reduced muscle mass) or obese patients. These and other evidence gaps must be resolved for the sake of future research.

These guidelines were drafted with reference to the “Minds Treatment Guideline Creation Companion 2014” using the Minds Guideline Creation support tool “GUIDE”. We would like to express our profound gratitude to Doctors Tsuguya Fukui and Takeo Nakayama of Minds for their roles as advisors in the creation of our guidelines.

We would also like to take this opportunity to express our appreciation to the many young physicians of the systematic review team for their contributions in drafting structured abstracts.

The primary significance of treatment guidelines is their application in daily clinical practice. We would appreciate any criticisms or ideas that would be useful in future revisions of these guidelines.

Shigeo Horie, M.D.

Professor and Chairman,

Department of Urology

Juntendo University, Graduate School of Medicine

## 2. On the Occasion of Publication

Cancer has been the leading cause of death among Japanese people for many years; currently, cancer is responsible for approximately 30% of all deaths in Japan. As the Japanese population ages, this figure will continue to increase year after year. Therefore, further development of treatment measures against cancer is undoubtedly one of the most crucial issues for the Japanese population. One such measure is drug therapy, which is widely performed. Many anticancer drugs are strongly associated with effects on various organs; a sufficient understanding of these associations is a prerequisite for effective and successful cancer drug therapy. Unfortunately, there have been no guidelines regarding cancer drug therapy in relation to associations with individual organs. Medical staffs and individuals involved in the treatment of cancer have a great interest for the relevance of the anti-cancer agent and a kidney. However, no previous guidelines exist that systematically described the association between cancer drug therapy and the kidneys.

In addition to chronic kidney disease, the concept of acute kidney injury has rapidly become widespread in recent years. As renal function assessment methods and biomarkers continue to develop, evolutions in nephropathy concepts are being observed.

Against this backdrop, the Japanese Society of Nephrology, the Japan Society of Clinical Oncology, the Japanese Society of Medical Oncology, and the Japanese Society of Nephrology and Pharmacotherapy have jointly published the “2016 Guidelines for the Treatment of Nephropathy in Cancer Pharmacotherapy”; the timely and fascinating publication of these guidelines marks a major step in the development of cancer pharmacotherapy. This is truly a document that individuals involved in cancer treatment have long awaited. I sincerely hope that this document will be used appropriately and effectively by all individuals who work on cancer treatment.

Lastly, I would like to express my deep gratitude to everyone involved in the drafting of these guidelines.

Seiichi Matsuo, MD. PhD.

President, Japanese Society of Nephrology

(President, Nagoya University)

As the Japanese population continues to age, physicians engaged in cancer pharmacotherapy increasingly encounter patients with organ dysfunction due to comorbid diseases; however, there is a lack of information regarding appropriate cancer pharmacotherapy for cancer patients with comorbid nephropathy. Currently, package inserts for the majority of anticancer drugs contain no clear information regarding administration in patients with chronic kidney disease. Although nephropathy is a major adverse event elicited by cancer pharmacotherapy, regimens for the prevention of nephropathy are currently modified based on the experience of individual physicians and the customs of individual facilities.

The present guidelines begin with renal function assessment methods necessary for determining doses of anticancer drugs, followed by descriptions of supportive therapy during cancer pharmacotherapy with cisplatin and other drugs for patients with decreased renal function. The guidelines also discuss supportive therapy for maintenance dialysis patients and patients with specific comorbidities. I believe that these guidelines will prove useful in daily clinical practice.

As part of its duties as a multidisciplinary academic society, the Japan Society of Clinical Oncology has been engaged in the formulation of guidelines for common supportive therapies for the treatment of cancer of various organs. Our society considers it greatly significant to have had the opportunity to participate in the formulation of these guidelines, which will contribute to improvements in the quality of treatment for patients with renal impairment.

Lastly, I would like to express my profound gratitude to Doctor Shigeo Horie, President of the Guideline Preparation Committee, for his tireless leadership in the drafting of these guidelines, as well as the many others who devoted their efforts to drafting the guidelines.

Yuko Kitagawa, M.D., Ph.D., F.A.C.S.

Chairman of Board of Directors, Japan Society of Clinical Oncology

Professor and Chairman, Department of Surgery, Keio University School of Medicine

Cancer is reported to afflict one in every two Japanese people and kill one in every three. As the Japanese population continues to age, the number of elderly cancer patients is likely to continue to increase. Consequently, an increase is also expected in the number of cancer patients with comorbidities such as nephropathy.

In the use of anticancer drugs for cancer patients with nephropathy, consideration must be given to the possibility of the enhancement of adverse events owing to diminished excretion, as well as the possibility that the toxicity of anticancer drugs will exacerbate nephropathy. However, as effective anticancer drugs are not used based solely on comorbid nephropathy, the therapy cannot be considered appropriate.

The performance of cancer pharmacotherapy in patients with nephropathy requires knowledge of not only oncology, but also nephrology. I believe that the joint creation of these guidelines by the Japanese Society of Nephrology, kidney specialists with the Japanese Society of Nephrology and Pharmacotherapy, and cancer therapy specialists with the Japan Society of Clinical Oncology is incredibly important and significant for the performance of appropriate pharmacotherapy in cancer patients with nephropathy. These guidelines establish crucial clinical questions and provide clear descriptions about these questions.

I anticipate that these guidelines will be utilized effectively by physicians, pharmacists, and nurses throughout Japan, and that they will be useful in the performance of appropriate anticancer drug therapy in cancer patients with nephropathy.

Yuichiro Ohe

National Cancer Center Hospital Department of Thoracic Oncology

The Japanese Society of Nephrology and Pharmacotherapy strives to foster “medical professionals who responsibly offer effective, safe, and the most appropriate drug therapy optimized to the individual patient”. Since the society was founded, it has worked toward fulfilling the following four major objectives: 1) to ensure the proper use of drugs and prevention of toxic side effects in patients with decreased renal function, 2) to prevent renal function deterioration and cardiovascular complications through proper medication guidance, 3) to provide appropriate drug therapy to dialysis patients with complications, and 4) to prevent drug-induced renal damage caused by nephrotoxic agents and drugs inducing renal ischemia. The Japanese Society of Nephrology and Pharmacotherapy was granted the opportunity to create the “2016 Guidelines for the Treatment of Nephropathy in Cancer Pharmacotherapy” alongside the Japanese Society of Nephrology, the Japan Society of Clinical Oncology, and the Japanese Society of Medical Oncology. The joint creation of these guidelines aligns with our own society’s goals, filling me with profound pride.

Similar to antibacterial agents and nonsteroidal anti-inflammatory drugs (NSAIDs), anticancer drugs can easily cause drug-induced nephropathy. The renal function of a patient receiving anticancer drugs fluctuates easily due to the effects of various factors such as the patient’s condition, activity level, and age. Anticancer drug pharmacokinetics, anticancer drug interactions, and conceptions of patients’ renal function are the fortes of our society, which specializes in nephrology and pharmacotherapy. In order to exert our specialized capacity, we recently established a Committee for the Formulation and Drafting of Guidelines. Going forward, with this committee at the center of our efforts, we hope to use our specialized perspective in relation to nephrology and pharmacotherapy to contribute to the drafting and revision of various types of practice and therapeutic guidelines.

In conclusion, I earnestly hope that the use of these guidelines will lead to the implementation of safer, more effective cancer drug therapy in all medical care settings through the prevention of anticancer drug-induced irreversible nephropathy, as well as the reduction and prevention of side effects, achieved by the establishment of appropriate dosages for patients with decreased renal function, including elderly patients.

Sumio Hirata,

President of the Japansese Society of Nephrology and Pharmacotherapy.

Professor and Director, Division of Clinical Pharmacology, Center for Clinical Pharmaceutical Sciences, Faculty of Pharmaceutical Sciences, Kumamoto University

## 3. Background

Nephropathy is a major potential adverse event in cancer drug therapy. Anticancer chemotherapy, particularly in patients with comorbid chronic kidney disease, requires sufficient examination of the balance of the potential therapeutic benefit with the risk of decreased renal function. However, cancer drug therapy in clinical settings has been performed based solely on physicians’ experience and instincts, a situation that calls for evidence-based guidelines.

The objective of the present guidelines was to draft clinical questions (CQs) and recommendations for those CQs to be specifically applied in real-world clinical practice. The overwhelming diversity of drugs used to treat cancer involves equally diverse nephropathy pathologies and dose adjustments. In establishing CQs, we have attempted be as comprehensive as possible. These guidelines take into account consistency with not only existing guidelines, but also guidelines on acute kidney injury treatment currently under production (Japanese Society of Nephrology, Japanese Society for Dialysis Therapy, Japan Society for Blood Purification in Critical Care, Japanese Society of Intensive Care Medicine, Japanese Society for Pediatric Nephrology, etc.).

In 2016, Japanese Society of Nephrology, Japan Society of Clinical Oncology, Japanese Society of Medical Oncology, and The Japanese Society of Nephrology and Pharmacotherapy established the Committee Of this guideline drafting group, which published Guidelines for treatment of renal injury during cancer chemotherapy 2016 in Jpn J Nephrol. 2016; 58:985-1050. This is the English version of that report. Chairman: Shigeo Horie.

## 4. Guideline objectives, assumed users, and social significance

This document includes guidelines regarding nephropathy in patients undergoing cancer drug therapy. These guidelines are intended to serve as a basis for assessing CQs that are likely to be frequently encountered in daily practice; they have been written for physicians, pharmacists, nurses, and all other medical personnel engaged in the treatment of cancer. The objective of the development of these guidelines was to support clinical assessments by obtaining answers as specific as possible regarding questions encountered in real-world practice by cancer specialists in order to convey current standard views and specifics of practice. However, we ultimately treat not cancer, but rather cancer patients; rather than performing individual medical acts uniformly, treatment should sufficiently respect each patient as an individual.

It is hereby specified that these guidelines do not contain assessment criteria for medical disputes or medical lawsuits.

## 5. Patients targeted by the guidelines

These guidelines are intended for the treatment of all adult cancer patients and not for pediatric cancer patients. The target of these guidelines is nephropathy directly caused by cancer drug therapy; the guidelines do not apply to, for example, nephropathy resulting from other causes in long-term cancer survivors.

## 6. Administrative framework

The drafting of these guidelines is characterized primarily by the participation of members from four different academic societies: the Japanese Society of Nephrology, the Japan Society of Clinical Oncology, the Japanese Society of Medical Oncology, and the Japanese Society of Nephrology and Pharmacotherapy. The drafting of these guidelines brought together nearly all of the principal groups currently engaged in cancer treatment and kidney disease in Japan, thereby allowing us to integrate all views currently standard in Japan. Furthermore, these guidelines were drafted in reference to the “Minds Treatment Guideline Creation Companion 2014” using the Minds Guideline Creation support tool “GUIDE”. Therefore, Doctors Tsuguya Fukui and Takeo Nakayama of Minds participated as advisors. We would like to take this opportunity to express out profound gratitude for their unerring advice to the drafting committee and their efforts in keeping our discussions focused.

## 7. Drafting method

First, the drafting committee formulated and listed 101 CQs, of which they adopted 16. For each CQ, we established keywords for literature searches. After performing a literature search, the systematic review team assessed each piece of literature, the guideline drafting committee made their recommendations and provided explanations for these choices, and the boards of each academic society approved these choices based on public comments in each of their societies.

## 8. Systematic review

We requested literature searches from the Japan Medical Library Association, on behalf of the systematic review team, with our searches open to all types of literature abstracted from the keywords. We searched for literature published from 1970 to 2014; the databases searched were PubMed, Ichushi-Web, and the Cochrane Library. Evidence was assessed in accordance with the Minds Treatment Guideline Creation Companion 2014 (Table 1). The systematic review team performed primary screening and secondary screening, and drafted an assessment sheet. All CQ database search results and literature assessment sheets were posted on each academic society’s website. Please feel free to refer to these posts as necessary.

**Table 1:** Assessment and definitions of overall evidence strength in systematic review

A (Strong): Strong confidence in effect estimates
B (Moderate): Moderate confidence in effect estimates
C (Weak): Limited confidence in effect estimates
D (Very Weak): Almost no confidence in effect estimates

## 9. Drafting of recommendations

Recommendation grades were determined based on the overall evidence assessments of the systematic review team with consideration for the trade-offs and balances between benefits and harm/side effects/risks. These recommendation grades were determined communally by the guideline drafting committee via informal consensus; the reasons underlying the committee’s assessments were recorded. Recommendation strength was rated on a scale of 1-4 as described below.

1. Strongly recommended
2. Weakly recommended (suggestion)
3. Weakly advised against (suggestion)
4. Strongly advised against

## 10. Outside assessment

These guidelines are posted on the websites of the four academic societies that collaborated to author them (the Japanese Society of Nephrology, the Japan Society of Clinical Oncology, the Japanese Society of Medical Oncology, and the Japanese Society of Nephrology and Pharmacotherapy); the guidelines were opened to public comments. All comments and our responses are posted on each society’s website. Following publication, these guidelines are scheduled to be assessed by the Appraisal of Guidelines for Research & Evaluation (AGREE) II instrument.

## 11. Issues in drafting of the guidelines

### 11.1 Assessment of renal function during cancer drug therapy

There is no established method for assessing renal function during cancer drug therapy. Although serum creatinine levels and eGFR, which are used to assess renal function in real-world clinical settings, are generally recognized to be somewhat problematic, there is currently no established method for assessing renal function before and after cancer drug therapy. The same is naturally true for proxy markers.

### 11.2 Diversity of anticancer drugs

The term “anticancer drug” covers an extremely large number of drugs. Each drug exerts different effects on renal function; discussing these individual effects is not the purpose of these guidelines. In order to introduce CQs frequently encountered in real-world cancer treatment, we have centered our discussion on widely used drugs. Wider varieties of cancer and drugs will be set aside as topics for future consideration.

### 11.3 Relationship to medical economics

For these guidelines, we did not examine issues in medical economics; therefore, the creation of the guidelines and the determination of recommendation levels were unaffected by concerns related to medical economics.

### 11.4 Reflection of patients’ opinions

It has been recommended that patients’ opinions be reflected in the creation of these guidelines. However, at the drafting stage, we were unable to construct a framework for incorporating patients’ opinions.

## 12. Sources of funding and conflicts of interest

All committee members involved in drafting these guidelines have submitted conflict of interest declarations in accordance with the regulations of their respective academic societies; these declarations are managed by each society’s secretariat. These guidelines have been drafted based purely on scientific grounds and assessment, as well as public interest. Individual committee members’ conflicts of interests associated with business-academia collaborations are managed properly in compliance with the Policy of Conflict of Interest in Clinical Research adopted by academic societies related to internal medicine.

The burden of funding the creation of the present guidelines was borne by the Japanese Society of Nephrology and the three related collaborating societies (the Japan Society of Clinical Oncology, the Japanese Society of Medical Oncology, and the Japanese Society of Nephrology and Pharmacotherapy). Funds were used for the drafting committee members’ transportation expenses, meeting site expenses, and meal expenses. These funds were not used for remunerations to the guideline drafting committee or the systematic review team.

## 13. Summary of guidelines

### 13.1 Assessment of renal function before and after cancer drug therapy

**CQ1: Is eGFR recommended for assessment of renal function for the adjustment of anticancer drug dosages?**

Recommendation grade: Weakly recommended (suggestion)

Recommendations

1. When assessing renal function for adjusting anticancer drug doses, eGFR is recommended if the patient’s condition is normal for their age and gender, i.e., if the patient is not malnourished, severely emaciated, or severely obese.
2. For patients whose muscle mass differs markedly from standard values due to malnourishment or severe emaciation, eGFR may not accurately reflect GFR. In such cases, rather than estimating GFR from serum Cr levels, combination with another method is recommended, such as measurement of GFR based on urine collection.
3. For drugs for which doses are fixed regardless of the patient’s condition, the dose should be adjusted in accordance with creatinine clearance (Ccr) or eGFR (mL/min) without correcting for body surface area.
4. For drugs for which the dose is determined by body surface area in accordance with the patient’s condition, it is reasonable to use Ccr corrected for body surface area (per 1.73 m^2^) or eGFR corrected for body surface area (mL/min/1.73 m^2^).
5. In the Cockcroft-Gault equation, Ccr (mL/min) is calculated using serum Cr values determined with the Jaffé method. When using Cr values determined with an enzymatic method, as is the standard in Japan, 0.2 is added to the actual Cr value.

Summary

When assessing renal function for adjusting anticancer drug doses, eGFR is recommended if the patient’s condition is normal for their age and gender, i.e., if the patient is not malnourished, severely emaciated, or severely obese. For patients whose muscle mass differs markedly from standard values due to malnourishment or severe emaciation, eGFR may not accurately reflect GFR. In such cases, rather than estimating GFR from serum Cr levels, combination with another method is recommended, such as measurement of GFR based on urine collection. For agents for which doses are fixed regardless of patient condition, the dose should be adjusted in accordance with creatinine clearance (Ccr) or eGFR (mL/min) without correcting for body surface area. For agents for which the dose is determined by body surface area in accordance with the patient’s condition, it is reasonable to use Ccr corrected for body surface area (per 1.73 m^2^) or eGFR corrected for body surface area (mL/min/1.73 m^2^). In the Cockcroft-Gault equation, Ccr (mL/min) is calculated using serum Cr values determined with the Jaffé method. When using Cr values determined with an enzymatic method, as is the standard in Japan, 0.2 is added to the actual Cr value.

Background and Objectives

In order to conduct anticancer chemotherapy safely and effectively, it is important to establish appropriate doses to elicit maximum anticancer effects and minimize side effects. When renal function is impaired, renally excreted drugs accumulate in the kidneys, potentially resulting in serious side effects; therefore, anticancer drug doses must be adjusted in accordance with renal function.

Estimated GFR is used to assess renal function. Outside of Japan, GFR is measured based on clearance of chromium (Cr) 51-labeled ethylenediaminetetraacetic acid and iodine (I)-125 sodium iothalamate, which are the respective GFR substances EDTA and iothalamate marked with radioisotopes of chromium and iodine, respectively [1]; in Japan, the gold standard is inulin clearance [1]. However, measurement of GFR requires urine collection following intravenous injection of exogenous clearance substances marked with inulin or radioactive material, thus making testing cumbersome. Therefore, Ccr and GFR are typically estimated based on serum Cr. Although various formulas have been devised for estimating GFR (Note 1) [2–8], most of these are intended for patients with chronic kidney disease and healthy individuals; their efficacy for cancer patients has not been sufficiently verified.

For patients with renal impairment, dose adjustments are often based on pharmacokinetic data at the time of clinical trials. Many trials of dose adjustment tailored to renal function have used Ccr as calculated from the Cockcroft-Gault equation. In 2010, the United States Food and Drug Administration (FDA) published guidance for pharmacokinetics research in patients with impaired renal function [9]. In addition to the conventional use of Ccr based on the Cockcroft-Gault equation, the guidance document also proposed the use of eGFR based on the Modification of Diet in Renal Disease (MDRD) equation; consequently, for drugs developed in the future, dose adjustments based on eGFR may become the norm. Proposed revised guidelines from the European Medicines Agency (EMA) also describe assessment of renal function using eGFR based on the MDRD equation and the Chronic Kidney Disease Epidemiology Collaboration (CKD-EPI) equation [10]. In Japan, the Guideline for Clinical Evaluation of Oral Hypoglycemic Agents published by the Ministry of Health, Labour and Welfare stipulates that renal function indicators (eGFR, Ccr, etc.) are recommended for assessment of clinical trials [11]. Although this guideline is not related to drug dose adjustments, it shows that eGFR may be used frequently to assess renal function in clinical trials in Japan going forward.

The objectives of this draft are to examine existing findings on renal function assessment in the administration of anticancer drugs, and to determine the usefulness and limitations of this assessment in real-world settings.

Commentary

Renal excretion of drugs occurs by glomerular filtration and tubular excretion; however, because there is no simple method for quantitatively assessing the drug excretion function of renal tubules, drug dose adjustments are typically based on GFR. Likewise, in the development of novel agents, doses are often established based on GFR or on Ccr, which reflects GFR. Therefore, GFR has been established as the reference for adjusting doses of anticancer drugs.

Measurement of GFR requires measurement of the clearance of a substance that is completely filtered by glomeruli, does not bind to proteins, is not metabolized in the body, and is not secreted or reabsorbed by renal tubules. In Japan, the gold standard is inulin clearance; other countries, however, measure clearance of substances such as 51Cr-EDTA, I-125 sodium iothalamate, or iohexol. Although Ccr is sometimes measured in place of GFR, measurement of Ccr (enzymatic method) yields values 20–30% higher than measurements of GFR based on inulin clearance. This discrepancy arises from the fact that Cr is not only filtered by glomeruli, but also secreted by renal tubules; consequently, GFR ≈ Ccr × 0.715 [12]. The use of these methods in clinical settings is constrained by the need for administration of reagents and urine collection, as well as a certain length of time before results are reported. These constraints have resulted in the development of equations for estimating GFR and Cr based on serum Cr levels.

Conventionally, drug doses have generally been adjusted using Ccr as estimated with the Cockcroft-Gault equation. However, because Ccr estimates are higher than GFR values, several different equations have been developed for the accurate estimation of GFR; these equations are now also used to adjust drug doses [13]. Most equations for calculating eGFR and Ccr were developed for use in healthy individuals and CKD patients; few such equations are intended for use in cancer patients. Although the Wright formula [5], the Martin formula [6], and the Jelliffe equation [7] are intended for the estimation of GFR in cancer patients, no method has been developed for estimating GFR specifically in Japanese cancer patients. Therefore, in regard to the CQ of whether eGFR is recommended for assessment of renal function for the adjustment of anticancer drug doses, we conducted literature searches upon establishing the following two questions: “Is eGFR based on serum Cr values an appropriate substitute for the gold standard of GFR based on clearance of inulin, 51Cr-EDTA, or I-125 sodium iothalamate?” and “Is eGFR an appropriate substitute for conventional Ccr calculated with the Cockcroft-Gault equation?” We found 12 studies that compared actual GFR to eGFR [14–25], three studies that compared actual Ccr to eGFR [26–28], and three studies that compared Ccr as calculated with the Cockcroft-Gault equation to formulas for eGFR and other such predictive formulas [29–31].

Results are inconsistent among studies that have examined the validities of various predictive formulas for cancer patients; this lack of consistency is assumed to potentially lead to the overestimation and underestimation of true GFR within a certain range. Overestimation of GFR can result in excessive doses of anticancer drugs and increased risk of side effects, while underestimation of GFR can lead to insufficient doses of anticancer drugs and a consequent attenuation of anticancer action. Few studies have compared actual GFR to eGFR as calculated with the Japanese Society of Nephrology’s equation in Japanese cancer patients; thus, further research is desirable. Research is also necessary to assess the usefulness of equations for estimating GFR based on serum cystatin C rather than serum Cr. Most studies compare eGFR to the gold standard of actual GFR; no studies have examined therapeutic effects and side effects resulting from administration of anticancer drugs based on eGFR. Research is also needed on clinical outcomes comparing the use of eGFR to the use of actual GFR or the use of Ccr as estimated with the Cockcroft-Gault equation.

At present, eGFR as calculated with the Japanese Society of Nephrology’s equation yields an approximate assessment of renal function; if renal function is normal, anticancer drug dose adjustment can be considered unnecessary. However, in the adjustment of doses based on data from clinical trials, it is safe to use the same renal function assessment methods and predictive equations. No matter which predictive equation is used, for patients with a markedly abnormal condition whose renal function necessitates anticancer drug dose adjustment or who are borderline for such adjustment, rather than using eGFR based on serum Cr value, it is safer to use a combination of other methods such as actual GFR based on urine collection (Note 2) and GFR as estimated based on cystatin C. Although actual GFR based on urine collection and inulin clearance is preferable, when these are difficult to implement, GFR can be approximated by multiplying Ccr (enzymatic method) by 0.715 [12].

When performing dose adjustments in accordance with Ccr or GFR, the following point must be noted: when assessing Ccr and GFR, the decision of whether to correct for body surface area is related to the method of measuring serum Cr value using the Cockcroft-Gault equation.

Drug doses are either fixed (mg/day) regardless of the patient’s condition (body weight and body surface area) or tailored to the patient’s condition (body weight and body surface area). In the use of agents for which the dose is fixed regardless of condition, the dose is adjusted in accordance with Ccr or eGFR (mL/min) without correcting for body surface area (Note 3). In regard to this point, the Japanese Society of Nephrology-edited 2012 CKD Practice Guide recommends the following: “When using renally excreted agents for patients with diminished renal function, renal function should be assessed with eGFR (mL/min) without correcting for body surface area, doses should be reduced, and administration intervals should be prolonged” [13]. The EMA Guideline on the evaluation of the pharmacokinetics of medicinal products in patients with decreased renal function also recommends that GFR be measured and recorded without correcting for body surface area [10]. On the other hand, in the use of agents for which doses are established based on body surface area (mg/m^2^) and body weight (mg/kg), it is reasonable to use Ccr corrected for body surface area (per 1.73 m^2^) or eGFR corrected for body surface area (mL/min/1.73 m^2^). Doing so is reasonable because when using Ccr or GFR per mL/min for correction in the use of agents for which the dose is adjusted in accordance with body surface area, the double-counting of patient condition leads to excessive doses for large-bodied patients and insufficient doses for small-bodied patients. Ccr values as calculated with the Cockcroft-Gault equation are in mL/min without correcting for body surface area, whereas in the MDRD equation and the Japanese Society of Nephrology eGFR equation, Ccr values are corrected per 1.73 m^2^ body surface area (mL/min/1.73 m^2^). Therefore, caution is necessary when applying these equations.

In Japan, Cr values are often measured with an enzymatic method; however, it must be noted that the Cockcroft-Gault equation uses Cr values determined with the Jaffé method. In the Jaffé method, Cr values are 0.2 mg/dL higher than Cr values determined with an enzymatic method; therefore, when calculating Cockcroft-Gault Ccr using Cr values determined with an enzymatic method, 0.2 is added to the enzymatic test Cr value.

Although some patients who undergo cancer drug therapy for urinary tract tumors possess only one kidney, eGFR reflects the aggregate function of both kidneys; therefore, eGFR can also be used for patients with only a single kidney.

* Note 1: Renal function estimation equations

1) Cockcroft-Gault equation [2]

$$ {\text{Estimated Ccr }}\left( {{\text{mL}}/{ \hbox{min} }} \right) \, = \, \left( { 1 40 - {\text{age}}} \right) \, \times {\text{ body weight }}\left( {\text{kg}} \right) \, \div \, \left( { 7 2 { } \times {\text{ serum Cr}}} \right) $$

For women, the above value is multiplied by 0.85. The serum Cr value is determined with the Jaffé method. For serum Cr values determined with an enzymatic method, 0.2 is added to the value.

2) Japanese Society of Nephrology eGFR equation [3]

$$ {\text{eGFR }}\left( {{\text{mL}}/{ \hbox{min} }/ 1. 7 3 {\text{ m}}^{ 2} } \right) \, = { 194 } \times {\text{ serum Cr}} - 1.0 9 4 { } \times {\text{ age}} - 0. 2 8 7 $$

For women, the above value is multiplied by 0.739.

3) MDRD equation [4]

$$ {\text{eGFR }}\left( {{\text{mL}}/{ \hbox{min} }/ 1. 7 3 {\text{ m}}^{ 2} } \right) \, = { 175 } \times {\text{ serum Cr}} - 1. 1 5 4 { } \times \, \left( {\text{age}} \right) \, - \, 0. 20 3 { } \times \, \left( {0. 7 4 2 { }\left[ {\text{for women}} \right]} \right) \, \times \, \left( { 1. 2 1 2 { }\left[ {\text{for black patients}} \right]} \right) $$

4) Wright formula [5]

$$ {\text{eGFR }}\left( {{\text{mL}}/{ \hbox{min} }} \right) \, = \, \left\{ {\left[ { 6 5 80 - \left( { 3 8. 8 { } \times {\text{ age}}} \right)} \right] \, \times {\text{ body surface area }} \times \, \left[ { 1- 0. 1 6 8 { } \times \, \left( {{\text{men }}0,{\text{ women 1}}} \right)} \right]} \right\} \, /{\text{ serum Cr}} $$

The serum Cr value is determined with the Jaffé method. See Note 2 for the formula for estimating body surface area.

5) Martin formula [6]

$$ {\text{eGFR }}\left( {{\text{mL}}/{ \hbox{min} }} \right) \, = \, \left\{ { 1 6 3 { } \times {\text{ body weight }}\left[ {\text{kg}} \right] \, \times \, \left[ { 1- \left( {0.00 4 9 6 { } \times {\text{ age}}} \right)} \right] \, \times \, \left[ { 1- 0. 2 5 2 { } \times \, \left( {{\text{men }}0,{\text{ women 1}}} \right)} \right]} \right\} \, /{\text{ serum Cr}} $$

6) Jelliffe equation [7]

$$ {\text{Estimated Ccr }}\left( {{\text{mL}}/{ \hbox{min} }/ 1. 7 3 {\text{ m}}^{ 2} } \right) \, = \, \left[ { 9 8- 1 6 { }\left( {{\text{age}} - 20} \right) \, /{ 2}0} \right] \, /{\text{ serum Cr}} $$

Used for patients aged 20-80 years. For women, the above value is multiplied by 0.9.

7) CKD-EPI equation [8]

$$ {\text{eGFR }}\left( {{\text{mL}}/{ \hbox{min} }/ 1. 7 3 {\text{ m}}^{ 2} } \right) \, = { 141 } \times \, \left( {{\text{serum Cr }}/ \, \kappa } \right) \, \alpha \, \times \, 0. 9 9 3 {\text{ age}} $$

κ is 0.9 for men and 0.7 for women.

α is −1.209 when serum Cr is larger than κ; otherwise, α is −0.411 for men and −0.329 for women.

For women, the above value is further multiplied by 1.018.

For black patients, the above value is further multiplied by 1.159.

Note: The unit of serum Cr values is μmol/L in the Wright formula and Martin formula, and mg/dL in all other equations

* Note 2: Measurement of actual GFR based on urine collection

**Fig. 1:**
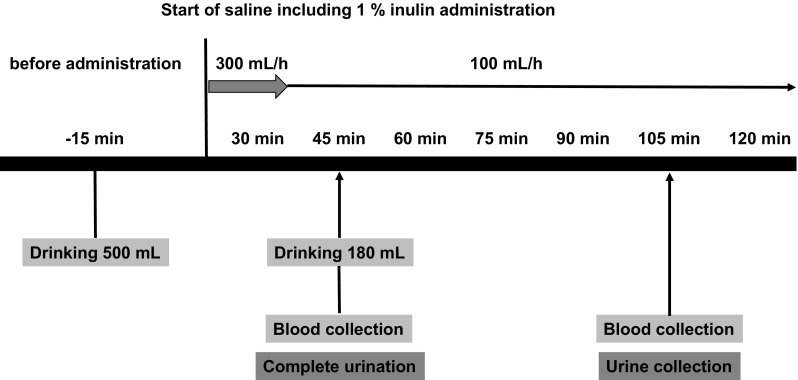
Simple inulin clearance method. 1) Complete urine collection 45 minutes after initiating inulin administration. Blood collection during urination. 2) Urine sampling upon urge to urinate at approximately 60 minutes of urine collection. Blood collection during urine sampling. 3) Accurate recording of urine collection time. 4) The blood concentrations of inulin in the two blood samples are averaged. Japanese Society of Nephrology [13]

When renal function must be assessed accurately, measurement of inulin clearance is recommended. There is a standard method and a simple method for doing so. In the standard method, saline solution containing 1% inulin is continuously infused; urine and midpoint blood are collected three times at 30-minute intervals, and the mean of the three clearances is calculated. In the simple method, urine is collected for approximately 1 hour under continuous infusion of inulin, and clearance is determined from blood collected before and after urine collection. The simple inulin clearance method is shown in a Fig. 1 [13]. Measurement of inulin clearance requires approximately 700 mL of additional fluid intake; thus, care must be taken to avoid excessive body fluid volume.

* Note 3: GFR not corrected for body surface area

Estimated GFR (mL/min/1.73 m^2^) predicts GFR for a standard body surface area; it does not represent actual GFR in individual patients. In patients whose condition differs greatly from the standard condition for their age and sex, eGFR overestimates or underestimates actual GFR. Therefore, when establishing drug doses, renal function must be assessed using GFR without correcting for body surface area (mL/min).

“Not correcting for body surface area” means determining the actual GFR of individual patients rather than correcting GFR units per 1.73 m^2^. Values yielded by estimation equations are already corrected per 1.73 m^2^ body surface area; therefore, GFR without correcting for body surface area is calculated as follows after determining the individual patient’s body surface area:

$$ {\text{GFR not corrected for body surface area }}\left( {{\text{mL}}/{ \hbox{min} }} \right) \, = {\text{ eGFR }}\left( {{\text{mL}}/{ \hbox{min} }/ 1. 7 3 {\text{ m}}^{ 2} } \right) \, \div { 1}. 7 3 { } \times {\text{ patients body surface area }}\left( {{\text{m}}^{ 2} } \right) $$

The DuBois formula [32], shown below, is a typical formula for estimating body surface area.

$$ {\text{Body surface area }}\left( {{\text{m}}^{ 2} } \right) \, = \, 0.00 7 1 8 4 { } \times {\text{ body weight }}\left( {\text{kg}} \right)^{0. 425} { } \times {\text{ height }}\left( {\text{cm}} \right)^{0. 725} $$

**CQ2: Is biomarker-based assessment recommended for early diagnosis of anticancer drug-induced AKI?**

Recommendation grade: Weakly recommended (suggestion)

Recommendation

At present, we cannot strongly recommend biomarker-based assessment for early diagnosis of anticancer drug-induced AKI. Although urine protein, urinary albumin, serum cystatin C, β2 microglobulin, urinary NAG, and urinary L-FABP can be measured as biomarkers of AKI in Japan, we cannot strongly recommend these or any other measurements as biomarkers of AKI.

Summary

In the last several years, several novel biomarkers of AKI have been reported. However, none of these biomarkers have yet demonstrated sufficient reliability, sensitivity, or rapidity in testing and assessment to be used in daily clinical practice.

Background and Objectives

1) Diagnostic criteria for AKI

In 2004, the Acute Dialysis Quality Initiative proposed the first uniform diagnostic criteria for AKI. These criteria focus on serum Cr and urine collection, which can be easily measured at any facility; the criteria are divided into 5 levels of renal dysfunction described by the acronym RIFLE (Risk, Injury, Failure, Loss, End-stage kidney disease) (Table 2) [33]. Furthermore, in 2007, the Acute Kidney Injury Network (AKIN) proposed its own classification (Table 3) [34]. The AKIN classification defines the diagnostic criteria for AKI as a 1.5-fold increase or an increase of ≥ 0.3 mg/dL in serum Cr within 48 hours, or reduced urinary output (< 0.5 mL/kg/h) over the span of 6 hours; severity is classified into 3 levels based on the degree of serum Cr increase and urinary output reduction. Severity is also assessed based on serum Cr increase and urinary output reduction within 1 week.

**Table 2:** Acute Dialysis Quality Initiative (ADQI) AKI diagnostic criteria (RIFLE classification). Crit Care. 2004;8:R204-12. (DOI 10.1186/cc2872) ©Bellomo R, et al.; licensee BioMed Central Ltd. 2004. http://ccforum.com/content/8/4/R204

	Diagnostic criteria based on serum Cr or GFR	Diagnostic criteria based on urine output (UO)
Risk	Increased serum Cr ≥ 1.5-fold the normal value, or GFR decrease > 25% normal value	UO < 0.5 mL/kg/h for 6 hours
Injury	Increased serum Cr ≥ 2-fold the normal value, or GFR decrease > 50% normal value	UO < 0.5 mL/kg/h for 12 hours
Failure	Increased serum Cr ≥ 3-fold the normal value, GFR decrease > 75% normal value, or serum Cr ≥ 4 mg/dL with acute rise ≥ 0.5 mg/dL	UO < 0.3 mL/kg/h for 24 h, or anuria for 12 hours
Loss	Need for renal replacement therapy for 4 weeks	
ESRD	Need for dialysis for longer than 3 months

**Table 3:** AKIN AKI diagnostic criteria (AKIN classification) Crit Care. 2007;11:R31. (DOI 10.1186/cc5713) ©Mehta RL, et al.; licensee BioMed Central Ltd. 2007

Stage	Diagnostic criteria based on serum Cr	Diagnostic criteria based on urine output (UO)
1	Increase in serum Cr ≥ 1.5-2-fold from baseline or ≥ 0.3 mg/dL	UO < 0.5 mL/kg/h for more than 6 hours
2	Increase in serum Cr ≥ 2-3-fold from baseline	UO < 0.5 mL/kg/h for more than 12 hours
3	Increase in serum Cr > 3-fold from baseline, or serum Cr ≥ 4 mg/dL with acute increase ≥ 0.5 mg/dL	UO < 0.3 mL/kg/h for 24 hours, or anuria for 12 hours

2) Background and objectives

Anticancer drugs frequently result in kidney injury; they are considered to account for 15% of all cases of drug-induced kidney injury, the third-leading cause of these injuries, following antibacterial agents and non-steroidal anti-inflammatory drugs [35]. Anticancer drugs are also known to result in AKI; AKI occurred in 36% of a group of 537 patients with acute myeloid leukemia or high-risk myelodysplastic syndrome who underwent induction chemotherapy, while 61.7% of patients who developed ESRD died [36]. A separate study reported an extremely high mortality rate of 73% among cancer patients with comorbid AKI [37]. Anticancer drug-induced AKI not only increases the risk of CKD and ESRD, but also requires adjustment of anticancer drug doses due to decreased renal function, thus interfering with the impending administration of the next anticancer drug. Due to the wide variety of chemotherapy regimens, AKI presents with a wide variety of clinical symptoms. Examples of anticancer drug-induced AKI are shown in Table 4 [38, 39]. A classic example of a tubular disorder-inducing drug is platinum-based agents, which primarily result in disorders in the tubulointerstitium. For example, cisplatin is known to cause AKI in approximately one-third of patients [40]. The anti-VEGF antibody bevacizumab is well known to cause vascular disorder and induce TMA.

**Table 4:** Examples of anticancer drug-induced AKI (includes only anticancer drugs covered by insurance in Japan) Kidney Int. 2015;87:909-17, Clin J A Soc Nephrol. 2012;7:1713-21.© [2012] Modified from the American Society of Nephrology

Renal vascular abnormalities	
Capillary leak syndrome	interleukin-2
TMA	bevacizumab, gemcitabine, cisplatin, mitomycin C, interferon
Glomerular abnormalities	
Minimal change disease	interferon, pemetrexed
Focal glomerulosclerosis	interferon, pemetrexed, zoledronic acid
Tubulointerstitial abnormalities	
Acute tubular necrosis	platinum-based agents, zoledronic acid, interferon, pentostatin, imatinib, pamidronate
Tubulitis (Fanconi syndrome)	cisplatin, ifosfamide, azacitidine, imatinib, pamidronate
Magnesium wasting	cisplatin, anti-EGFR monoclonal antibodies
Nephrogenic diabetes insipidus	cisplatin, ifosfamide, pemetrexed
Syndrome of inappropriate antidiuretic hormone secretion	cyclophosphamide, vincristine
Acute interstitial nephritis	sorafenib, sunitinib
Tubular obstructive nephropathy	methotrexate

Although the emergence of the RIFLE and AKIN classifications, which are based on serum Cr and urinary output, have resulted in significant advances in the diagnosis of AKI, many issues remain. Serum Cr is affected by several factors such as age, body weight, sex differences, other agents, muscle metabolism, protein intake, and hypervolemia; thus, it is deeply flawed as a biomarker of AKI [41, 42]. In addition, elevated serum Cr does not manifest until 48-72 hours after the initial occurrence of nephrotoxicity, thus hindering prompt AKI diagnosis and therapeutic intervention [42]. To compensate for the flaws of serum Cr, the usefulness of many novel biomarkers of AKI has been examined. However, the clinical use of novel biomarkers of AKI still faces high hurdles due to the need to establish threshold values in accordance with sex differences, age differences, and primary diseases [43].

The objectives of this guideline are to examine the latest findings regarding biomarkers for AKI induced by anticancer drugs, and to determine the usefulness and limitations of these biomarkers in real-world clinical settings.

Commentary

The biomarkers discussed in this draft can be assessed objectively and serve as indicators of pharmacological responses to biological changes, histological changes, and therapeutic interventions [44]. Biomarkers of anticancer drug-induced AKI must be immune to interference from all types of treatment. Potential roles for biomarkers include: 1) risk assessment, 2) early diagnosis, 3) classification of disease stage, 4) differential diagnosis, 5) indication of therapeutic effects, and 6) determination of prognosis. Anticipation is particularly high for the practical application of biomarkers that enable earlier diagnosis than do serum Cr and eGFR.

The present draft divides biomarkers into two categories: those that can be used in clinical practice and are covered by health insurance in Japan, and those that cannot. Also, in 2010, the Predictive Safety Testing Consortium’s Nephrotoxicity Working Group submitted results for drug toxicity studies and analyses of biomarker performance to the FDA and the European Medicines Evaluation Agency; these results presented Kidney Injury Molecule-1 (Kim-1), urinary albumin, urine protein, β2 microglobulin, serum cystatin C, clusterin, and trefoil factor 3 (TFF-3) as biomarkers related to renal function safety [45]. Although the objective of this report is limited to safety assessments, we felt it necessary to discuss the usefulness of the above 7 biomarkers as biomarkers of anticancer drug-induced AKI.

## 1. Biomarkers for which measurement is covered by health insurance

### a) Urinary albumin

Urinary albumin levels increase as a result of enhanced glomerular permeability and impaired proximal tubular reabsorption. In fact, short-term and long-term administrations of nephrotoxic anticancer drugs have been reported to increase levels of urinary microalbumin [46]. However, urinary albumin levels are known to increase not only as a result of AKI, but also due to factors such as fever, exercise, dehydration, diabetes, and hypertension; thus, the specificity of urinary albumin as a biomarker of AKI is considered limited [47].

### b) Urine protein

In detection of glomerular disease, urine protein is said to be superior to BUN and serum Cr in terms of diagnostic performance [48]; however, urine protein is reported to have low specificity as a biomarker of AKI [49], and its usefulness has not been established.

### c) Serum cystatin C

Cystatin is the most important cysteine protease inhibitor in the human body. Cystatin C, a 13-kDa protein secreted by all nucleated cells, is characterized by the fact that it does not bind to plasma proteins. Therefore, cystatin C is freely filtered by the renal glomeruli; after being reabsorbed by the proximal tubules, more than 99% of it is degraded by the endocytic receptor megalin [50]. Unlike Cr, cystatin C is not secreted by the renal tubules into urine, and its levels are not dependent on sex or muscle mass. In patients with mild to moderate renal impairment, serum cystatin C is well correlated with GFR [51]; thus, cystatin C can be used to detect nephrotoxicity at an early stage with greater sensitivity than serum Cr, thus making serum cystatin C a potentially useful biomarker of AKI [52]. However, serum cystatin C is limited in the following two ways: 1) it is affected by diabetes, high levels of corticosteroids, hyperthyroidism, inflammation, hyperbilirubinemia, and hypertriglyceridemia [53]; 2) when GFR reaches < 15 mL/min/1.73 m^2^, the increase in serum cystatin C slows and levels off at 5-6 mg/L [54]. Benöhr et al. [55] have demonstrated that serum cystatin C levels are significantly elevated on day 5 following cisplatin administration compared to 3 days prior to administration. At present, serum cystatin C has not been established as a useful biomarker of anticancer drug-induced AKI. Although measurement of serum cystatin C is covered by health insurance, measurement of urinary cystatin C is not.

### d) β2 microglobulin

β2 microglobulin is a polypeptide comprising 99 amino acids with a molecular weight of 11,800; it is distributed on the surface of nucleated cells throughout the body as the L chain of the major histocompatibility complex HLA class I antigen. β2 microglobulin passes freely through the glomerular basement membrane and is almost completely reabsorbed by the proximal tubule; in tubular disorders, however, decreased reabsorption leads to increased excretion of β2 microglobulin in urine, thus making β2 microglobulin a potentially useful marker of AKI. In fact, urinary β2 microglobulin has been reported to increase 4-5 days earlier than does serum Cr in tubular disorders [56]. However, in aciduria and at room temperature, β2 microglobulin is extremely unstable, thus limiting its usefulness as a biomarker [57].

### e) NAG

In the kidneys, NAG is a glycolytic enzyme present in lysosomes and produced in the endoplasmic reticula of proximal tubule cells. Tubular disorders result in increased excretion of NAG in urine, thus making urinary NAG a potentially useful marker of AKI; urinary NAG is reported to demonstrate abnormal values 12 hours to 4 days earlier than does serum Cr [58]. Goren et al. [59] compared concentrations of NAG before and after cisplatin administration in 12 patients. In their investigation, concentrations of NAG increased following cisplatin administration, reached their peak on day 3, and subsequently decreased. In an examination of NAG and β2 microglobulin in 8 patients before and after cisplatin administration, Ikeda et al. [60] reported that β2 microglobulin reached peak levels on day 3 and decreased to pretreatment levels in 1 week, although only 1 patient demonstrated increased NAG for 2 weeks. However, urinary NAG activity is inhibited by many nephrotoxic substances, magnesium, and endogenous urea [61]. Furthermore, urinary NAG levels are increased not only in AKI, but also in rheumatoid arthritis [62], impaired glucose tolerance [63], and hyperthyroidism [64]; thus, the specificity of urinary NAG for AKI is considered low.

### f) Urinary L-FABP

Liver fatty acid-binding protein is a fatty acid transport protein that is expressed in the proximal tubule and that possesses antioxidant properties [65]. Human L-FABP possesses a hypoxia-inducible factor 1a responsive element; thus, L-FABP expression is induced by hypoxia [66]. Tubular disorders are known to result in increased excretion of L-FABP into urine; patients who develop AKI following cardiovascular surgery are reported to demonstrated an increase in urinary L-FABP immediately after surgery [67], while a high urinary L-FABP value is reported to be an independent predictor of AKI [68]. As a biomarker of AKI, L-FABP compares favorably with Kim-1, NGAL, and NAG [69]. In Japan, assessment of L-FABP for the diagnosis of AKI is covered by health insurance. However, there has been very little investigation of the usefulness of L-FABP as a biomarker of anticancer drug-induced AKI in humans; further study is necessary going forward.

## 2. Biomarkers for which measurement is not covered by health insurance

### a) Urinary Kim-1

Kidney Injury Molecule-1 is a transmembrane glycoprotein produced in the proximal tubule during kidney injury; for 12 hours following renal ischemia, excretion of the extracellular domain of Kim-1 into urine is increased [70]. In animal models of cisplatin-induced nephrotoxicity, levels of Kim-1 increased earlier than did levels of serum Cr, indicating that Kim-1 is a useful biomarker of tubular disorders [71]. In addition, a systematic review reported fluctuation of Kim-1 within 24 hours of kidney injury [72]. The United States FDA has approved Kim-1 as a marker of AKI. Tekce et al. [73] compared levels of serum Kim-1 and urinary Kim-1 prior to cisplatin administration and at days 1, 3, and 5 after cisplatin administration in 8 patients with AKI and 14 patients without AKI with an eGFR ≥ 90 mL/min. On day 1, there were no significant differences between the groups in serum Cr, eGFR, or serum Kim-1; however, urinary Kim-1 levels were significantly higher in the AKI group. On day 3, the two groups demonstrated significant differences in serum Cr, eGFR, and urinary Kim-1; however, there were no significant differences in serum Kim-1. Thus, urinary Kim-1 demonstrates potential as an early marker of cisplatin-induced AKI. However, the stability of Kim-1 is markedly reduced in urine; thus, further study of urinary Kim-1 is considered necessary [74].

### b) NGAL

Neutrophil gelatinase-associated lipocalin (NGAL) is a 25-kDa glycoprotein secreted primarily by activated neutrophils; under normal circumstances, 100% of NGAL is reabsorbed by the proximal tubule. In tubular disorders, NGAL is expressed in the ascending limb of the loop of Henle and in part of the collecting ducts; due to increased excretion into blood and urine, NGAL demonstrates abnormal values 2-4 hours following AKI. A meta-analysis of more than 2,500 cases found that NGAL is a useful marker not only for the diagnosis of AKI, but also for renal prognosis [75]. Peres et al. [76] reported that following the administration of cisplatin, the group of patients with AKI demonstrated higher NGAL levels than the non-AKI group; however, this difference was not significant. Gaspari et al. [77] also compared NGAL levels between a group of 12 AKI patients and a group of 12 non-AKI patients at 1 and 4 hours and at 1, 2, 3, 7, and 15 days after cisplatin administration. Although a significant difference between the AKI group and the non-AKI group in serum Cr was first observed on day 3 following cisplatin administration, a significant difference in NGAL was first observed on day 1. Therefore, NGAL may enable detection of cisplatin-induced AKI earlier than does serum Cr.

### c) Clusterin

Clusterin, a 76-80-kDa glycoprotein, is assumed to exert an anti-apoptotic renoprotective effect in kidney injury. Urinary clusterin is reported to be superior to BUN and serum Cr in the detection of proximal tubular injury [48]. However, insufficient research has been done on clusterin in regard to human AKI, and the usefulness of clusterin as a biomarker of anticancer drug-induced AKI is unknown.

### d) Urinary TFF-3

Urinary excretion of TFF-3 is reduced in AKI. Although urinary TFF-3 has been demonstrated to be a useful marker of AKI in animal models, its usefulness in humans has not been sufficiently examined [78].

### e) Endothelin-1

Endothelin-1 is a 21-amino acid protein that possesses a vasoconstrictor effect; in the kidneys, it is expressed in mesangial cells and collecting ducts. Takeda et al. [79] measured urinary endothelin-1-like immunoreactivity/Cr before and 1 and 2 weeks after cisplatin treatment; these authors reported that urinary endothelin-1-like immunoreactivity/Cr was significantly increased at 1 and 2 weeks after cisplatin treatment compared to pretreatment levels. Following cisplatin treatment, β2 microglobulin/Cr and endothelin-1-like immunoreactivity/Cr peaked on day 2 and subsequently declined, whereas NAG/Cr peaked on day 6.

In addition to the above, other substances have also been examined for their usefulness as biomarkers of AKI, such as interleukin-18, angiotensinogen, tissue inhibitor of metalloproteinase-2, and insulin-like growth factor-binding protein 7 [65].

The usefulness of various biomarkers has been examined in animal models [80]; however, there is a significant dearth of evidence in humans. The following circumstances account for the near-absence of evidence: 1) The lack of uniformity in diagnostic criteria for AKI leads to differences among reports. 2) Although many reports have examined AKI overall, very few reports have focused on anticancer drug-induced AKI. 3) For many biomarkers, evident threshold values have not been established, making assessment in individual studies difficult. 4) Cancer drug therapy often combines multiple drugs. Different drugs induce nephrotoxicity via different mechanisms, while some drugs (cisplatin, etc.) are assumed to act via multiple mechanisms; therefore, assessment with a single biomarker may not be valid (in fact, a study has demonstrated the usefulness of a combination of multiple biomarkers [49]). 5) When using serum, the possibility cannot be ruled out that what is being assessed is not AKI, but rather the effects of anticancer drugs throughout the body. Furthermore, in such cases, the effects of other factors, such as age and past history of CKD and other complications, are unknown.

Although biomarkers enhance our understanding of drug-induced AKI, much remains unknown regarding their contribution to the diagnosis of AKI. When conducting anticancer chemotherapy, nephrologists must determine when biomarkers are necessary, which biomarkers are useful, how to interpret biomarker data, and how to utilize biomarker data on an individual basis in treatment for each patient.

### 13.2 Prevention of decreased renal function during cancer drug therapy

(1) Overview

**CQ3: Is reduction of anticancer drug doses recommended for mitigating toxicity in patients with decreased renal function?**

Recommendation grade: Weakly recommended (suggestion)

Recommendation

When using drugs that lead to an increased risk of adverse drug events in patients with decreased renal function, dose reduction is recommended. However, when the goal is to cure cancer, doses must ultimately be determined with consideration of the balance between risks and benefits.

Summary

When using agents that lead to an increased risk of adverse drug events in patients with decreased renal function, dose reduction is recommended. However, when the objective is to cure cancer, doses must ultimately be determined with consideration of the balance between risks and benefits.

Background and Objectives

The kidneys are an elimination pathway for many anticancer drugs and their metabolites; therefore, renal impairment can delay the excretion and metabolism of anticancer drugs, potentially resulting in increased toxicity and thus necessitating consideration of dose reduction [81]. For patients with decreased renal function, dose reduction is also sometimes considered for anticancer drugs that are metabolized in the liver. For example, dose reduction is considered necessary when irinotecan is administered to dialysis patients [82–85]. For sorafenib, as well, a drug that is primarily metabolized in the liver, some believe that dose reduction should be considered [86]. The present draft summarizes evidence related to dose reduction and presents principles for dose reduction for major anticancer drugs.

Commentary

Answering CQ3 requires studies comparing frequencies of adverse drug events between normal doses and reduced doses in patients with decreased renal function; however, the search formula used in the present guidelines yielded no relevant literature. Such studies present ethical issues and are considered difficult to conduct. Much of the available evidence [87–90] comes from studies that compared the frequencies of adverse drug events in patients with normal renal function and patients with decreased renal function (reduced doses) [87–90]. However, there are very few such studies; thus, the quality of the evidence is judged to be extremely low (D: Almost no confidence in effect estimates).

Consideration of the balance between benefits and risks is particularly important in determining recommendation levels, but due to the paucity of evidence regarding the efficacy of treatment with reduced doses, our recommendation is weak.

However, in real-world clinical settings, attempts have been made to reduce doses in accordance with renal function and to control plasma drug concentrations; these attempts have yielded a small number of studies and guidelines that serve as references. One such attempt with carboplatin dosing is the Calvert formula, which calculates doses using target AUC and Ccr as estimated with the Cockcroft-Gault equation based on the results of a phase I clinical trial (see CQ10 for details) [91]. Another study has reported a revised Calvert formula based on data from Japanese patients [92].

Although there are no comprehensive guidelines regarding dose reduction methods in Japan, the Japanese Society of Nephrology and Pharmacotherapy [93] has presented opinions on dose reduction methods for several anticancer drugs (Table 5); in addition, there are various books with information on dose reduction for anticancer drugs [94]. Outside of Japan, the United States FDA [95] and the European Medicines Agency [96] have published guidelines calling for the inclusion of methods of administration for patients with decreased renal function in package inserts for all types of drugs; these publicly available package inserts may also serve as a reference for dose reduction.

**Table 5:**
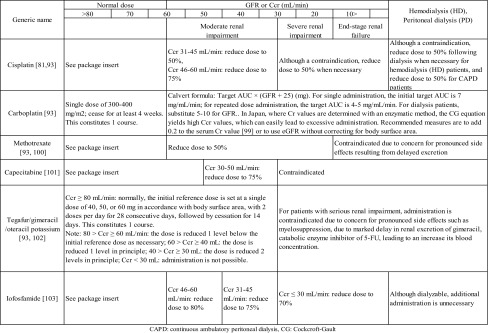
Dose reduction methods for major anticancer drugs in patients with decreased renal function

For anticancer drugs, the therapeutic range and the toxic range are extremely close to each other. Therapeutic drug monitoring is considered useful for preventing toxicity in such cases; in fact, therapeutic drug monitoring has been proven effective in randomized clinical trials for some anticancer drugs [97, 98]. However, at present, attempting to measure blood concentrations of anticancer drugs is not standard practice.

A realistic desirable approach for patients with decreased renal function is to begin anticancer drug administration by referring to the above-mentioned dose adjustment guidelines, monitor adverse events more closely than normal, and consider adjusting doses in future treatment. In patients for whom the objective is to cure cancer, doses must ultimately be determined in consideration of the balance between risks and benefits.

(2) Platinum-based drugs

**CQ4: Is risk factor assessment recommended for predicting cisplatin-induced AKI?**

Recommendation grade: Weakly recommended (suggestion)

Recommendation

Reported predictors of cisplatin-induced AKI include hypoalbuminemia; smoking; female sex; age (1.03-fold increase in risk per year of age); concomitant use of other anticancer drugs; comorbid cardiovascular disease or diabetes; advanced cancer; and total cisplatin dose. In order to prevent cisplatin-induced AKI, risk factors should be assessed prior to drug administration.

Summary

Reported predictors of cisplatin-induced AKI include hypoalbuminemia; smoking; female sex; age (1.03-fold increase in risk per year of age); concomitant use of other anticancer drugs; comorbid cardiovascular disease or diabetes; advanced cancer; and total cisplatin dose. However, among existing studies, there is no consistent definition of AKI, there are no clear threshold values for risk factors, and there are no established measures for cases with risk factors. Thus, many issues remain for further investigation.

Background and Objectives

Cisplatin, a key drug in treatment for many types of cancer, is one of the most commonly used anticancer drugs. However, cisplatin is known to produce side effects such as myelosuppression, intestinal toxicity, and neurotoxicity; another crucial side effect, nephrotoxicity, is a potential subsequent cisplatin dose-limiting factor. One-third of patients receiving cisplatin are presumed to have comorbid AKI [104], which often results in the limitation of subsequent doses of cisplatin. Furthermore, AKI sometimes develops into chronic tubulointerstitial fibrosis and irreversible chronic tubulopathy, which may further progress to CKD [105, 106]. The present draft examines risk factors that may serve as predictors of cisplatin-induced AKI.

Commentary

Cisplatin-induced renal injury is considered to manifest primarily as proximal tubular injury, particularly in the S3 segment [107]. Cisplatin is absorbed from the basolateral surface into cells and injures mitochondrial DNA, thereby activating apoptosis. Intracellular accumulation of cisplatin results in inflammation, oxidative stress, and ischemic injury [105]. Hypomagnesemia is also considered to cause renal injury. Magnesium is thought to be involved in active transport mechanisms in the renal tubules. Sobrero et al. have supposed that hypomagnesemia leads to an increased concentration of cisplatin in renal tubular cells, thereby causing proximal tubular injury [108].

In an investigation by de Jongh et al. [109] of weekly-dose cisplatin for 400 patients with locally advanced or metastatic cancer, 36% of patients received cisplatin alone, 49% received cisplatin + etoposide, and 15% received cisplatin + paclitaxel. A total of 116 patients (29%) demonstrated a reduction in Ccr of ≥ 25%, while 29 patients (7%) were unable to continue cisplatin due to nephrotoxicity. Independent predictors of post-cisplatin nephrotoxicity as determined by multivariate analysis were paclitaxel coadministration (odds ratio [OR] 4.0, p = 0.001), hypoalbuminemia (OR 3.5, p = 0.006), smoking (OR 2.5, p = 0.002), female sex, and old age. According to age group, the risk of nephrotoxicity was 26% among patients aged < 48 years, and increased with age to 35% for patients aged 48-62 years and 41% for patients aged > 62 years; the risk of nephrotoxicity increased 1.03-fold per year (OR 1.03, p = 0.007). Regarding gender, the risk of nephrotoxicity was twice as high for women as for men (OR 2.0, p = 0.025). Another study reported that cisplatin excretion capacity is lower in women than in men [110]; however, the cause of this difference is unknown. The involvement of smoking in nephrotoxicity has been surmised to be the effect of oxidant stress [111]; however, one possibility that cannot be ruled out is that smoking causes cardiovascular disease, which secondarily leads to post-cisplatin nephrotoxicity. Also, in hypoalbuminemia, an increased concentration of unbound cisplatin is considered to enhance nephrotoxicity [109]. The cited study, which defines nephrotoxicity as a reduction in Ccr of ≥ 25%, is not strictly an assessment of predictors of AKI.

In an investigation of 425 patients treated with cisplatin (total dose 220 mg/m^2^ [median]), Stewart et al. [112] reported that in multivariate analysis, the factors that predicted maximum increases in serum Cr up to 4 weeks after cisplatin treatment were serum albumin, serum potassium, body surface area, and number of administrations. However, this study contains flaws: renal function was assessed with serum Cr alone, and the authors’ method of assessing maximum increases in serum Cr up to 4 weeks after cisplatin treatment is neither a well-established method nor period for assessment. Furthermore, anticancer drugs were used in combination with many other drugs; thus, the degree to which cisplatin contributes to changes in renal function is unknown.

In an examination of 1,721 patients treated with cisplatin, Mizuno et al. [113] found, in multivariate analysis, that cancer stage 4 diagnosis (OR 1.8, p = 0.011) and total cisplatin dose were risk factors for moderate AKI (1.5-1.9-fold increase in serum Cr within 7 days of cisplatin treatment), while comorbid cardiovascular disease, comorbid diabetes mellitus, and cancer stage 4 diagnosis were risk factors for severe AKI (≥ 2.0-fold increase in serum Cr within 7 days of cisplatin treatment).

Several studies have thus reported predictors of AKI. However, these studies do not present a consistent definition of AKI, and no studies have utilized the RIFLE or AKIN classifications. Furthermore, there are no clear threshold values for risk factors, and there are no established measures for cases with risk factors. Thus, many issues remain for further investigation.

**CQ5: Are divided doses of cisplatin recommended for preventing nephrotoxicity?**

Recommendation grade: Strongly advised against

Recommendation

Divided doses of cisplatin are not recommended for preventing nephrotoxicity, as the significance of this practice has not been established.

Summary

Divided doses of cisplatin are not recommended for preventing nephrotoxicity, as the significance of this practice has not been established.

Background and Objectives

The fact that the kidneys are the primary organs that excrete platinum-based agents, particularly cisplatin, is related to the nephrotoxicity induced by these agents; this nephrotoxicity is considered to be caused by tubular necrosis. Nephrotoxicity is often prevented or alleviated by hydration via large-volume fluid replacement, or by administration of magnesium. Although some physicians prefer to use divided doses of platinum-based agents to prevent or alleviate nephrotoxicity, some studies with pediatric cancer patients have reported that nephrotoxicity is less frequent with continuous drug administration than with divided doses. The present draft examines the recommendation level for the current practice of administering divided doses of cisplatin with the intention of alleviating nephrotoxicity.

Commentary

At present, there are no articles detailing prospective randomized clinical trials on divided doses of platinum-based agents with alleviation of nephrotoxicity as the primary endpoint. No studies have directly examined the nephrotoxicity prevention effect of divided doses in adult subjects, while there are only three observation studies that have compared divided doses of cisplatin to other administration methods. The content and results of these studies are summarized below.

Forasteiere et al. compared 5 divided, intermittent doses of cisplatin (each administered over 20 minutes) to the same total dose administered via continuous infusion (24 hours) [114]. The subjects were patients with head and neck cancer; 6 patients received cisplatin 30 mg/m^2^ via continuous infusion (24 hours) for 5 days, while another 5 patients received cisplatin 30 mg/m^2^ via intermittent intravenous bolus (20 minutes) for 5 days; the two groups were compared in terms of total platinum concentration, free platinum concentration, and adverse events. Although the continuous infusion group demonstrated an extremely low maximum unbound platinum concentration compared to the intermittent bolus group, the exposure to unbound platinum (AUC) was 1.5-2 times higher in the continuous infusion group. An assessment of subclinical nephrotoxicity based on measurement of urinary excretion of the renal enzymes NAG and alanine found no differences between the two groups in nephrotoxicity, or in hearing loss or nausea/vomiting. In contrast, myelosuppression and hypomagnesemia were observed frequently in the continuous infusion group, suggesting that total platinum exposure contributes to nephrotoxicity more than does peak concentration. Because adverse events in continuous administration were clinically acceptable, the authors recommended larger-scale trials with continuous infusion of cisplatin. However, because this study compared two forms of divided doses against each other, the merits of divided doses remain unknown.

Ikeda et al. investigated the optimal administration method for combined 5-FU + cisplatin therapy in patients with gastric cancer and esophageal cancer [115]. The study compared pharmacokinetic differences (AUC and C_max_) in 12 courses of therapy for 9 subjects. Comparisons were made among three groups: 4 courses of cisplatin 80 mg/m^2^ (2 hours), 4 courses of 20 mg/m^2^ (2 hours) for 5 days, and 4 courses of 100 mg/m^2^ (120 hours). In all three groups, 5-FU was continuously infused at a dose of 800 mg/m^2^ (24 hours) for 5 days. The authors concluded that continuous infusion is the pharmacokinetically optimal administration method; however, this method has not been recognized to be superior in terms of adverse events.

Takahashi et al. also compared pharmacokinetics and nephrotoxicity according to different cisplatin administration methods (5 divided doses, 24 hours continuous infusion, 12 hours continuous infusion, 6 hours continuous infusion); they found no differences in clinical adverse events [116].

The above-cited three studies found no difference in nephrotoxicity based on the cisplatin administration method; thus, there is no basis for the active recommendation of divided doses. Therefore, due to the current absence of appropriately designed studies, there is no basis for actively recommending divided doses of cisplatin for the prevention of nephrotoxicity. On the other hand, the National Comprehensive Cancer Network’s 2014 bladder cancer guidelines state that divided doses (35 mg/m^2^ on days 1 and 2 or days 1 and 8) may be considered for patients with borderline renal function or minimal dysfunction [117]. However, no references are cited for these proposed divided doses. In addition, the therapeutic effect of divided doses is unclear.

Nonetheless, some believe that continuous administration of cisplatin is safe for preventing and alleviating nephrotoxicity. Erdlenbruch et al. compared pharmacokinetics and nephrotoxicity between two groups: a group of 4 pediatric osteosarcoma patients who received continuous infusion of 120 mg/m^2^ cisplatin over 72 hours, and a group of 6 pediatric medulloblastoma patients who received 1-hour bolus infusions of 40 mg/m^2^ cisplatin per day for 3 consecutive days [118]. The divided dose group demonstrated a peak concentration of free platinum 19 times that of the continuous infusion group, as well as a lower minimum GFR and a higher rate of persistent nephrotoxicity within 1 year after the completion of cisplatin therapy. The authors concluded that continuous administration of cisplatin is less nephrotoxic than divided doses.

**CQ6: Is hydration (≥3 L/day) during cisplatin administration recommended for mitigating nephrotoxicity?**

Recommendation grade: Strongly recommended

Recommendation

Hydration (≥3 L/day) during cisplatin administration is recommended for mitigating nephrotoxicity.

Summary

The nephrotoxicity of cisplatin was established at the preclinical level (animal trials); therefore, cisplatin dosage regimens were formulated from the outset using hydration and other forms of supportive therapy. Consequently, despite the absence of high-quality evidence from sources such as randomized clinical trials, hydration is strongly recommended during cisplatin administration.

Background and Objectives

Platinum-based agents are renally excreted anticancer agents used to treat various forms of cancer; these agents are well known to be nephrotoxic. Cisplatin is particularly nephrotoxic and has thus been examined frequently. The primary measures for preventing cisplatin nephrotoxicity are hydration and administration of diuretics. The issues of fluid replacement volume and the use versus non-use of diuretics are covered in another CQ and are thus not discussed here.

Commentary

Answering the present CQ fundamentally requires a randomized clinical trial examining the use versus non-use of hydration during cisplatin therapy in human subjects; however, our search formula did not retrieve any such trials. Most of the existing relevant literature consists of reviews that discuss nephrotoxicity. As a basis for recommending fluid replacement, one typical review [119] cites an animal trial [120] that divided dogs into a control group, a prehydration group, and a mannitol infusion group; nephrotoxicity was alleviated in the latter two groups. Many other reviews have also recommended forced diuresis with hydration and diuretics. Cisplatin was developed in the 1970s; as a result of the different development methodologies of today, as well as the fact that cisplatin was known early in its development to be nephrotoxic, the absence of validation studies in human subjects is considered inevitable. Consequently, the quality of the evidence is assessed as extremely poor (D: Almost no confidence in effect estimates).

In the evidence for various existing cancer drug therapies, implementation plans prescribe normal hydration when using cisplatin. For other platinum-based agents (carboplatin, etc.), hydration is not typically prescribed. The Japanese package insert for cisplatin states in the dosage section that hydration is to be performed before, during, and after administration; however, the package insert for carboplatin does not contain such instructions. In the United States as well, the package insert for cisplatin calls for hydration, whereas the package insert for carboplatin does not (rather, it specifies that, unlike with cisplatin, massive hydration and forced diuresis are normally not to be performed).

In consideration of the above information, and of the balance between benefits and risks, hydration during cisplatin administration is strongly recommended. Hydration is not recommended during administration of other platinum-based agents such as carboplatin. In the past, hydration during cisplatin administration commonly consisted of approximately 2 L saline solution or half-normal saline solution prior to cisplatin and ≥ 1 L saline solution or half-normal saline solution after cisplatin. In regard to “short hydration”, which reduces this hydration volume and uses oral rehydration, please see CQ7.

**CQ7: Is short hydration recommended during cisplatin administration?**

Recommendation grade: Weakly recommended (suggestion)

Recommendation

When administering cisplatin on an outpatient basis, short hydration is recommended with consideration of renal function, performance status (PS), and age. However, performing short hydration safely requires sufficient oral rehydration and establishment of sufficient urinary output. Short hydration is intended for patients who, from day 0 to day 3 of chemotherapy, can consume a normal amount of food and undergo an additional ~1,000 mL of hydration per day. When oral rehydration is insufficient, it is necessary to modify the environment to enable rapid hydration via intravenous infusion.

Summary

When administering cisplatin, short hydration is recommended with consideration of renal function, PS, and age. Performing short hydration safely requires sufficient oral rehydration; short hydration is intended for patients who, from day 0 to day 3 of chemotherapy, can consume a normal amount of food and undergo an additional ~1,000 mL of hydration per day. When oral rehydration is insufficient, the environment must be modified to enable rapid hydration via intravenous infusion. In addition, short hydration requires establishment of sufficient urinary output via diuretics (mannitol or furosemide), supplementation of magnesium and potassium, and confirmation of serum electrolyte levels.

Background and Objectives

Before and after administration of cisplatin, hydration must be performed in order to prevent nephrotoxicity. In Japan, standard practice is to replace 1,000-2,000 mL fluid over the course of ≥ 4 hours before and after cisplatin and to administer cisplatin diluted with ≥ 500-1,000 mL infusion solution over the course of ≥ 2 hours. However, this hydration is performed over a long period of time and requires hospitalization. A number of studies have examined methods of hydration for preventing cisplatin-induced nephrotoxicity. Here, we have examined the safety of short hydration via 2,000-2,500 mL fluid replacement over the course of approximately 4 hours.

Commentary

In 2007, Tiseo et al. [121] reported the results of a retrospective two-center observational study regarding the safety of high-dose cisplatin (≥ 75 mg/m^2^) administered with short hydration. Following administration of approximately 2,000 mL saline solution and furosemide over the course of 4 hours on the day of cisplatin administration, nephrotoxicity resulted in withdrawal of chemotherapy in 5 of 107 subjects (4.6%); among these 5 subjects, 2 demonstrated Grade 2 nephrotoxicity according to National Cancer Institute Common Toxicity Criteria. In Japan, Horinouchi et al. [122] and Hotta et al. [123] have conducted small-scale prospective trials with patients who received 75 mg/m^2^ and 60 mg/m^2^ cisplatin, respectively. With short hydration incorporating potassium, magnesium, and mannitol, elevations in serum Cr of Grade 2 or higher (based on the reference range upper limit in the Common Terminology Criteria for Adverse Events ver. 4.0) occurred in 2.2% (1/44) and 0% (0/46) of subjects, respectively. In all other literature we assessed [124–130], compared to conventional hydration, short hydration was found not to increase the incidence of nephrotoxicity and was concluded to be safe; the results of these studies were judged to be consistent. The short hydration method assessed in the present CQ is as follows: a total of approximately 1,600-2,500 mL fluid is replaced over the course of approximately 4 hours; potassium and magnesium are supplemented; and urinary output is established via diuretics (furosemide and mannitol). In contrast, the United States National Comprehensive Cancer Network presents a chemotherapy order template of a total of 1,000-3,000 mL hydration before and after cisplatin administration at a rate of 250-500 mL/h for many carcinomas [131]. In Japan, the Japan Lung Cancer Society and the Japanese Society of Medical Oncology have created a guide that mentions short hydration, stating that short hydration can be performed safely if the target patients are limited to those who meet certain criteria [132]. These target patients fulfill conditions such as the following: age < 75 years, serum Cr value below the center’s reference value, Ccr ≥ 60 mL/min, PS of 0-1 on the Eastern Cooperative Oncology Group scale, no pleural effusion or ascites, cardiac function capable of withstanding approximately 500 mL/h hydration (left ventricular ejection fraction ≥ 60% on echocardiography, etc.), completion of appropriate antiemetic therapy, and ability to receive approximately 1,000 mL/day oral hydration from day 0 to day 3 of cisplatin administration. Therefore, consciousness of disease and assurance of adherence are important when selecting patients. In addition, in the event of serious side effects or insufficient water intake, fluid replacement should be performed at a center that can adapt rapidly to such circumstances. All of the target studies were observational studies; therefore, the quality of the overall evidence was initially graded as C (weak). There were judged no serious problems with risk of bias, indirectness, inconsistency, imprecision, or publication bias, all of which downgrade the quality of evidence. In addition, intervention effects, dose-response gradient, and confounders, all of which improve the quality of evidence, were judged not to apply; therefore, the overall quality of evidence was ultimately graded as C (weak).

**CQ8: Are diuretics recommended for preventing cisplatin-induced nephrotoxicity?**

Recommendation grade: Weakly recommended (suggestion)

Recommendation

We cannot definitively recommend diuretics for preventing cisplatin-induced nephrotoxicity. Such a preventive effect has not been proven in small-scale randomized clinical trials; therefore, there is no basis for recommending diuretics to achieve this effect. Nevertheless, diuretics are used to prevent nephrotoxicity during cisplatin treatment, which has been widely performed since the 1970s. The efficacy and safety of diuretics for preventing nephrotoxicity have been confirmed in large-scale clinical trials of cisplatin and other therapies. Therefore, there is also no basis for rejecting the use of diuretics to prevent cisplatin-induced nephrotoxicity.

Summary

We cannot definitively recommend diuretics for preventing cisplatin-induced nephrotoxicity. Such a preventive effect has not been proven in small-scale randomized clinical trials; therefore, there is no basis for recommending diuretics to achieve this effect. Nevertheless, diuretics are used to prevent nephrotoxicity during cisplatin treatment, which has been widely performed since the 1970s. The efficacy and safety of diuretics for preventing nephrotoxicity have been confirmed in large-scale clinical trials of cisplatin and other therapies. Therefore, there is also no basis for rejecting the use of diuretics to prevent cisplatin-induced nephrotoxicity.

Background and Objectives

High-dose cisplatin administration was first reported to be possible with the combined use of hydration and diuretics in the 1970s. Since then, the osmotic diuretic mannitol and the loop diuretic furosemide have been used to prevent nephrotoxicity in the administration of cisplatin. Here, we examine whether these diuretics are effective for preventing cisplatin-induced nephrotoxicity.

Commentary

A phase I clinical trial for cisplatin has demonstrated that nephrotoxicity is a dose-limiting factor [133]. Hydration and diuretics have been used in attempts to rapidly excrete toxic platinum metabolites and reduce their duration of contact with the renal tubule in order to alleviate nephrotoxicity. However, pharmacokinetic analysis has shown that diuretics do not affect the half-life of free platinum following cisplatin administration and are considered to reduce the urinary excretion rate and increase serum platinum concentration [134, 135]. Even if diuretics are effective in preventing nephrotoxicity, the mechanism of this effect is not sufficiently understood.

Hayes et al. were the first to report that fluid replacement and mannitol alleviate cisplatin-induced nephrotoxicity. High-dose cisplatin (120 mg/m^2^) was administered to 60 patients with the combined use of hydration and mannitol. The 52 patients who were analyzed demonstrated only temporary increases in serum Cr; no serious nephrotoxicity was observed. Serum Cr increased by < 2 mg/dL in almost all patients; although 10 patients demonstrated greater increases, 9 of those patients demonstrated reduced renal function at baseline and were at high risk for nephrotoxicity [136]. Following this report, diuretics have been used in the majority of subsequent clinical trials for therapies that include cisplatin.

Ostrow et al. conducted the first trial comparing mannitol and furosemide. Twenty-two patients with advanced cancer resistant to existing therapies received 100 mg/m^2^ cisplatin. The subjects were assigned to one of two groups; one group received 37.5 g mannitol by 6-hour infusion, while the other group received 40 mg furosemide by intravenous injection 60 minutes prior to treatment. All subjects underwent hydration with 1 L normal saline following cisplatin administration. Nephrotoxicity (defined as Ccr ≤ 50 mL/min or serum Cr > 2 mg/dL) occurred in 28% of the 22 courses in the mannitol group and 19% of the 25 courses in the furosemide group. Mean Ccr values in the mannitol group and the furosemide group were 34 mL/min and 26 mL/min, respectively. Although the mannitol group demonstrated a tendency toward more severe nephrotoxicity, the difference was not statistically significant. Therefore, the interpretation is that neither diuretic was demonstrated to be superior [137].

In a prospective randomized phase II trial, Al-Sarraf et al. compared the incidence of post-cisplatin nephrotoxicity between hydration alone and hydration + mannitol. Nephrotoxicity occurred following initial cisplatin administration in 30% of patients who received hydration only and in 15% of patients who received hydration + mannitol. The overall incidence of nephrotoxicity in the hydration only group and the hydration + mannitol group was 39% and 32%, respectively. Thus, mannitol demonstrated a prevention effect against cisplatin-induced nephrotoxicity in the initial administration of cisplatin; however, this effect was not evident in subsequent administrations [138].

Santoso et al. conducted a randomized comparative trial to compare the cisplatin-induced nephrotoxicity prevention effects of hydration (500 mL normal saline), hydration + furosemide (40 mg), and hydration + mannitol (50 g). Forty-nine patients with gynecologic cancers underwent therapy with 75 mg/m^2^ cisplatin + paclitaxel or 5-FU and were randomly assigned to one of the three above-described combination therapies. A total of 15 women were assigned to the hydration only group, while 17 were assigned to the hydration + furosemide group and 17 were assigned to the hydration + mannitol group. The three groups demonstrated nearly equal Ccr at baseline. However, following cisplatin therapy, Ccr (± standard deviation) in the hydration only group, the hydration + furosemide group, and the hydration + mannitol group was 80.4 (±33.5), 81.4 (±23.3), and 60.6 (±26.8) mL/min, respectively; thus, the hydration + mannitol group demonstrated a result significantly inferior to that of the other two groups [139]. Several issues have been highlighted in relation to this trial: the trial was discontinued due to poor outcomes in the hydration + mannitol group, the sample size was small, the dose of mannitol was larger than in previous trials, and urine collection for Ccr lacked rigor, among other issues. Thus, despite being a randomized comparative trial, its quality is lacking.

Therefore, although diuretics have been widely used since the 1970s to prevent nephrotoxicity induced by platinum-based agents, no randomized comparative studies have clearly demonstrated that diuretics are effective to that end, and there is no sufficient basis to recommend diuretics for that purpose. The European Society of Clinical Pharmacy Special Interest Group on Cancer Care states in their recommendations on the prevention of cisplatin nephrotoxicity that there is no reason to recommend the use of diuretics [140]. However, diuretics have been widely used in the administration of cisplatin for many years, and this approach has been used to create evidence for various therapies; thus, the safety of diuretics in cisplatin administration is well established. In short hydration as well, which has been attempted in recent years, the use of diuretics is presumed, and they have been reported to be safe. Therefore, without proof that diuretics pose evident risks, there is little basis to advise against the use of these agents.

**CQ9: Is magnesium recommended for preventing cisplatin-induced nephrotoxicity?**

Recommendation grade: Weakly recommended (suggestion)

Recommendation

Administration of magnesium, which can be expected to prevent hypomagnesemia, has been indicated to affect renal function favorably; therefore, administration of magnesium is recommended for preventing nephrotoxicity.

Summary

Prophylactic administration of magnesium can be expected to prevent hypomagnesemia and alleviate nephrotoxicity following the administration of cisplatin.

Background and Objectives

Due to enhanced excretion primarily from the kidneys as well as intestinal toxicity, cisplatin administration frequently results in hypomagnesemia, which has been reported to potentially cause nephrotoxicity. Therefore, prophylactic administration of magnesium is anticipated to alleviate nephrotoxicity.

Commentary

In our searches for studies comparing nephrotoxicity in the use versus non-use of magnesium in patients receiving high-dose cisplatin, we found two randomized comparative trials and one retrospective analysis [141–143].

Willox et al. randomly assigned 17 cancer patients (16 patients with testicular cancer and 1 patient with an ovarian dysgerminoma) scheduled to receive cisplatin into a magnesium treatment group and a non-magnesium treatment group; the non-magnesium group subsequently demonstrated significant tubular damage (high NAG value) [141]. Bodnar et al. conducted a double-blind, randomized comparison of magnesium administration and non-administration (placebo) in ovarian cancer patients scheduled to receive cisplatin; the magnesium group subsequently demonstrated significantly favorable GFR compared to the placebo group [142]. Although both of the above studies indicate that prophylactic administration of magnesium affects renal function favorably, the sample sizes were small, and the endpoints and statistical hypotheses were unclear. Therefore, although magnesium can be anticipated to prevent nephrotoxicity, this preventive effect has not been definitively verified.

However, prophylactic administration of magnesium is inferred to prevent hypomagnesemia and consequently alleviate adverse reactions such as nephrotoxicity, while adverse reactions induced by prophylactic administration of magnesium are minor; considering these points, prophylactic administration of magnesium is currently recommended.

**CQ10: Is carboplatin dose setting based on renal function recommended?**

Recommendation grade: Strongly recommended

Recommendation

In adult cancer patients receiving carboplatin, there is insufficient evidence to prove that the method of setting doses based on renal function following the establishment of a target AUC increases therapeutic effects and reduces side effects compared to the general method of determining doses based on body surface area. However, the setting of doses based on renal function is both reasonable and widespread in daily clinical practice.

Summary

In adult cancer patients receiving carboplatin, there is insufficient evidence to prove that the method of setting doses based on renal function following the establishment of a target AUC increases therapeutic effects and reduces side effects compared to the general method of determining doses based on body surface area. However, the setting of doses based on renal function is both reasonable and widespread in daily clinical practice. Therefore, we graded our recommendation as “strong”.

Background and Objectives

The platinum-based agent carboplatin is almost completely excreted from the kidneys following administration; therefore, its pharmacokinetics can be predicted based on GFR. Furthermore, AUC, an indicator of drug exposure volume in the body, is closely correlated with hematotoxicity and antitumor effect. Consequently, it is now widespread practice to set carboplatin doses based on GFR after establishing a target AUC. In many cases, GFR is substituted by Ccr. The present draft examines the validity of the routine clinical practice of setting carboplatin doses based on renal function.

Commentary

Carboplatin is a platinum-based agent with a broad antitumor spectrum that primarily includes gynecologic cancer and lung cancer. Because carboplatin is almost completely excreted from the kidneys following administration, its pharmacokinetics can be predicted based on GFR [144]. Furthermore, AUC, an indicator of drug exposure volume in the body, is strongly correlated with thrombocytopenia and other forms of hematotoxicity, which limit carboplatin doses; AUC is also strongly correlated with antitumor effect. Therefore, individual differences in the side effects and therapeutic effects of carboplatin can be explained by individual differences in AUC arising from pretreatment GFR [145]. Setting carboplatin doses based on GFR after establishing a target AUC minimizes individual differences in AUC, which can consequently be expected to reduce the risks of serious hematotoxicity and undertreatment. Based on this idea, Calvert et al. created a formula for setting carboplatin doses based on GFR; this formula, called the Calvert formula, remains widely used in daily clinical practice today [146].

$$ {\text{Calvert formula}}:{\text{ dose }}\left[ {\text{mg}} \right] \, = {\text{ target AUC }}\left[ {{\text{mg}}/{\text{mL }} \times { \hbox{min} }} \right] \, \times \, \left( {{\text{GFR }}\left[ {{\text{mL}}/{ \hbox{min} }} \right] \, + { 25}} \right) $$

To calculate doses, the previously established target AUC and the patient’s GFR are entered into the Calvert formula. Based on clinical trials, AUC is set at 5-7; however, analysis of a model for ovarian cancer patients demonstrates that while the antitumor effect of carboplatin nearly plateaus at an AUC of 5-7, thrombocytopenia and other forms of hematotoxicity are enhanced as AUC increases [147]. Similar formulas for calculating carboplatin doses based on renal function have been created by Egorin et al. [148, 149] and Chatelut et al. [150]; however, the Calvert formula remains the most widely used due to its simplicity. In any case, setting dosages based on renal function is reasonable. However, no clinical trial has prospectively examined the setting of doses based on renal function from the perspective of increasing therapeutic effects and reducing side effects in comparison to typical methods based on body surface area; thus, the evidence for setting doses base on renal function is insufficient.

In the process of creating the Calvert formula, the investigators employed actual GFR based on clearance of EDTA labeled with ^51^Cr, a radioisotope of chromium. In Japan, the gold standard for GFR is inulin clearance; although measurement of inulin clearance is covered by health insurance, the procedure is cumbersome, and thus, Ccr is often used instead in daily clinical practice. However, not only does serum Cr undergo glomerular filtration, approximately 20-30% of it is secreted from the renal tubules; consequently, Ccr yields higher values than does GFR, a point which requires caution. The serum Cr values used to calculate Ccr are measured with the Jaffé method and an enzymatic method. The Jaffé method is affected by non-specific substances in serum; therefore, the measured value of serum Cr is approximately 0.2 mg/dL higher than the true serum Cr value. However, in calculations of Ccr, this measurement error is cancelled out by the difference with GFR resulting from tubular secretion; therefore, in effect, Ccr calculated with serum Cr as determined by the Jaffé method approximates GFR. With an enzymatic method, on the other hand, serum Cr measurements are precise, and Ccr values are higher than GFR values. Consequently, the Calvert formula, which uses Ccr as a substitute for GFR, engenders a risk of excessive carboplatin dosing. Since the mid-1990s, most medical centers in Japan have used the enzymatic method, whereas the United States and Europe have used the Jaffé method until recently. Caution is necessary when interpreting clinical trials of carboplatin conducted outside Japan. One proposed measure is to add 0.2 to enzymatic method-based serum Cr values when calculating Ccr [151, 152]. When GFR is used, it is calculated with the Japanese Society of Nephrology’s GFR estimation formula (eGFR) without correcting for body surface area (see CQ1). However, many Japanese clinical trials currently use Ccr calculated from enzymatic method-based serum Cr values in place of GFR. When evidence from these trials is used in clinical practice, regardless of the presence of evident bias between the actual AUC and the target AUC, what is effectively being used is GFR estimated with the same methods as in the relevant trials. However, considering the objective of individualized patient doses based on renal function, renal function should be accurately assessed from the clinical trial stage in order to prevent bias between actual AUC and target AUC.

In the United States, since 2010, serum Cr has been measured by isotope dilution mass spectrometry, which are as accurate as the enzymatic method. Accordingly, establishing an upper limit for the GFR used in the Calvert formula (125 mL/min) is recommended to avoid the excessive carboplatin dosing that would result from overestimation of renal function. In gynecology, for extremely low serum Cr, a lower limit (0.7 mg/dL) is sometimes established. With these methods, it must be noted that actual AUC is larger than target AUC in the majority of patients, while actual AUC is smaller than target AUC in some patients with favorable renal function.

The “GFR+25” component of the Calvert formula corresponds to total carboplatin clearance; “GFR” corresponds to renal clearance, while the constant “25” corresponds to non-renal clearance. Non-renal clearance depends primarily on an individual’s physical size. The Calvert formula was created in the United Kingdom; when using the Calvert formula for Japanese individuals, who are physically smaller on average than Caucasians, the proportion of non-renal clearance increases relative to GFR, particularly in patients with severely decreased renal function, thereby potentially leading to excessive carboplatin dosing [153].

Measurement of Ccr requires urine collection (typically for 24 hours); thus, estimates calculated based on serum Cr values are sometimes used as a substitute for GFR. Formulas for estimating Ccr include the Cockcroft-Gault equation and the Jelliffe equation. Formulas for estimating GFR in the United States and Europe include the MDRD equation, the CKD-EPI equation, and the Wright formula; for Japanese patients, the previously described GFR estimation formula (eGFR) is used (see CQ1). When using these formulas, the patient’s background (e.g., racial differences and pathologies) and the serum Cr measurement method must be noted. Furthermore, these formulas presume that serum Cr is stable; when renal function fluctuates markedly (such as in the acute phase of renal failure) or when muscle mass is greatly reduced (such as in sarcopenia or undernutrition), renal function will be overestimated.

(3) Other agents

**CQ11: Is urine alkalinization recommended for preventing nephrotoxicity in high-dose methotrexate therapy with leucovorin rescue?**

Recommendation grade: Strongly recommended

Recommendation

Urine alkalinization is recommended for preventing nephrotoxicity in methotrexate with leucovorin rescue.

Summary

In methotrexate with leucovorin rescue, in addition to urine alkalinization and diuresis via sufficient hydration, monitoring of blood methotrexate levels is recommended. In addition, increased doses and prolonged administration of leucovorin in accordance with serum methotrexate levels are recommended.

Background and Objectives

Methotrexate with leucovorin rescue was developed in the 1970s; supportive therapies such as urine alkalinization and diuresis via sufficient hydration were generally established by the 1990s. The present draft re-examines this method based on recent findings.

Commentary

More than 90% of methotrexate is excreted from the kidneys. In animal experiments, methotrexate nephrotoxicity has been shown to arise from the accumulation of methotrexate or its metabolite 7-OH-MTX in the renal tubules. The solubility of methotrexate and its metabolites depends on urinary pH; this solubility is considered to increase five- to eight-fold with an increase in pH from 6.0 to 7.0 [154]. Methotrexate with leucovorin rescue was developed in the 1970s with the following theoretical basis: when high-dose (generally ≥ 500-1000 mg/m^2^) methotrexate is administered, the methotrexate is passively incorporated into cancer cells; after a certain amount of time has elapsed, leucovorin is administered as a methotrexate antidote and is passively incorporated by healthy cells capable of doing so, thereby rescuing those cells. Methotrexate with leucovorin rescue has been demonstrated as effective against osteosarcoma, acute leukemia, and malignant lymphoma; however, in the 1970s, the frequency of drug-related deaths was at a high rate of approximately 6% [155]. The pathological explanation for these drug-related deaths emphasized the following: methotrexate nephrotoxicity results in delayed excretion of methotrexate itself, thereby aggravating myelosuppression and other serious adverse events [155]. Subsequently, in addition to diuresis with urine alkalinization [156] and sufficient hydration [157], monitoring of blood methotrexate levels has become widespread, as have increased doses and prolonged administration of leucovorin based on serum methotrexate levels [158]. In accordance with these technical improvements, deaths related to methotrexate with leucovorin rescue have decreased; data for 3,887 cases of osteosarcoma aggregated in 2004 showed a rate of deaths related to methotrexate with leucovorin rescue of 0.08% [159]. Based on the above, although there is no evidence from randomized comparative trials, the establishment of urinary output through urine alkalinization and sufficient hydration are recommended to prevent nephrotoxicity in methotrexate with leucovorin rescue. However, in the above-cited 2004 data, nephrotoxicity Grade ≥ 2 (WHO criteria, serum Cr levels 1.5-3.0 × upper limit of normal) was observed in 68 patients (1.8%); the methotrexate with leucovorin rescue-related mortality rate among these patients was 4.4%, thus remaining high [159]. Increased doses of leucovorin are reported to be effective for delayed excretion of methotrexate resulting from nephrotoxicity [160].

The efficacy of recombinant enzymes for directly degrading methotrexate in plasma has recently been reported in a prospective trial [161] and a retrospective analysis [162]; these recombinant enzymes have been approved in the United States, but not in Japan. Methotrexate is a small molecule with a molecular weight of only 454.44 and thus can be removed by hemodialysis. On the other hand, approximately 50% of methotrexate binds to proteins, and its volume of distribution is tens of liters; in 4 hours, hemodialysis removes only 10.8% of methotrexate (according to drug information). However, the use of high-flux membranes in hemodialysis has been reported in case studies to remove methotrexate more efficiently [163, 164], thus making this technique worthy of consideration as a therapeutic approach.

On the other hand, in combination chemotherapy that includes standard-dose methotrexate, i.e., cyclophosphamide, methotrexate, and 5-FU (CMF) for breast cancer and methotrexate, vinblastine, doxorubicin, and cisplatin (M-VAC) for urothelial carcinoma, there is no definitive evidence showing that leucovorin and urine alkalinization are useful for preventing nephrotoxicity. In addition, the combined use of non-steroidal anti-inflammatory drugs in combination chemotherapy that includes standard-dose methotrexate is reported to exacerbate adverse events; therefore, methotrexate should not be used in combination with non-steroidal anti-inflammatory drugs [154].

**CQ12: Is withdrawal or reduction of angiogenesis inhibitors recommended when proteinuria is observed?**

Recommendation grade: Strongly recommended

Recommendation

When proteinuria is observed during administration of angiogenesis inhibitors, withdrawal or reduction of these drugs is recommended upon consideration of the grade of proteinuria and of the risks/benefits of continued drug therapy.

Summary

Administration of angiogenesis inhibitors requires regular measurement of blood pressure, urinalysis for early detection of hypertension and proteinuria, and proactive administration of antihypertensive agents for sufficient control of blood pressure. If proteinuria manifests, temporary withdrawal of angiogenesis inhibitors or continued treatment with reduced doses are reasonable options; however, in the case of grade 1 proteinuria, for patients with advanced cancer, another option is to continue treatment upon consideration of the risks and benefits. When proteinuria is grade 2 or higher, angiogenesis inhibitors are temporarily withdrawn or reduced, and the patient is treated by a nephrologist as necessary.

Background and Objectives

Angiogenesis inhibitors, which are clinically applied in the treatment of various carcinomas, inhibit tumor angiogenesis primarily by suppressing the VEGF pathway. The actions and adverse events of angiogenesis inhibitors differ from those of cytotoxic anticancer drugs. Proteinuria, like hypertension, is an adverse event that occurs during treatment with angiogenesis inhibitors [165]. Proteinuria and microalbuminuria have been demonstrated to be independent risk factors for renal disease and cardiovascular disease [166]; thus, when proteinuria manifests during administration of angiogenesis inhibitors, appropriate management is necessary. There are many different types of angiogenesis inhibitors, each of which is indicated for a different carcinoma and has a different treatment regimen. Angiogenesis inhibitors are administered upon initiation of drug therapy for carcinomas that in most cases occur in a solitary kidney, such as advanced renal cell carcinoma. Furthermore, angiogenesis inhibitors are sometimes administered alone and also sometimes used as part of multidrug therapy. With this diverse background, the incidence of proteinuria during administration of angiogenesis inhibitors has been determined to differ for each individual agent [165]. According to Japanese special drug use surveillance, during the administration of bevacizumab in 2,696 cases of advanced colorectal cancer, proteinuria occurred in 4.60% of cases; proteinuria was serious in 0.11% of these cases [167]. The incidence of proteinuria during the administration of sunitinib in 2,141 cases of advanced renal cell carcinoma and gastrointestinal stromal tumors was 1.59%, versus an incidence of 1.20% in advanced renal cell carcinoma and 2.98% in gastrointestinal stromal tumors [168]. During the administration of sorafenib in 3,335 cases of advanced renal cell carcinoma, the incidence of proteinuria was 0.71%, with no cases of serious proteinuria reported [169]. In a phase II clinical study of 64 Japanese patients with cytokine-refractory metastatic renal cell carcinoma, proteinuria occurred in 58% of patients, 9% of whom developed serious proteinuria of grade 3 or higher [170].

Commentary

Angiogenesis inhibitors, i.e., VEGF pathway inhibitors, result in proteinuria during treatment; although the precise mechanism of onset of this adverse effect has not been determined, the presumed mechanism is a breakdown of glomerular structure and filtration function originating from the inhibition of VEGF production by podocytes [171]. Angiotensin-converting enzyme inhibitors and ARBs dilate efferent arterioles, reduce intraglomerular pressure, and reduce proteinuria; therefore, administration of angiogenesis inhibitors must involve regular measurement of blood pressure, urinalysis for early detection of proteinuria, and proactive administration of antihypertensive agents for sufficient control of blood pressure [165].

While the incidence of proteinuria is different for each individual angiogenesis inhibitor, the risk of proteinuria is considered to be dose-dependent [172, 173]. Thus, when proteinuria manifests, reduction or temporary withdrawal of angiogenesis inhibitors are practical options. In fact, in a clinical trial that investigated the therapeutic effects of various molecularly targeted agents, many patients who demonstrated grade 2 or higher proteinuria during treatment were able to resume treatment following dose reduction or withdrawal [174]. When advanced cancer patients with limited outcomes develop grade 1 proteinuria during treatment with angiogenesis inhibitors, withdrawal or reduction is not always necessary; rather, the decision must be based on an examination of the benefits/risks of continued drug therapy and on the individual patient’s wishes. However, cases of nephrotic syndrome have been confirmed during treatment with various types of angiogenesis inhibitors [175–177]. In cases in which proteinuria worsens despite temporary withdrawal or reduction of angiogenesis inhibitors, referral to a nephrologist should be considered [165].

**CQ13: Is reduction of bisphosphonates and anti-RANKL antibodies recommended for patients with decreased renal function?**

Recommendation grade: Strongly recommended

Recommendation

Reduction of bisphosphonates is recommended for patients with decreased renal function. However, reduction of anti-RANKL antibodies is not recommended for patients with decreased renal function.

Summary

Reduction of bisphosphonates is recommended for patients with decreased renal function. However, reduction of anti-RANKL antibodies is not recommended for patients with decreased renal function.

Background and Objectives

Injectable bisphosphonates have been established as useful for improving malignancy-induced hypercalcemia and inhibiting skeletal-related events associated with bone lesions resulting from multiple myeloma or bone metastases from solid tumors (defined as pathologic fracture, radiotherapy for bone lesions, surgery for bone lesions, spinal cord compression, and hypercalcemia). The primary bisphosphonates used in treatment for malignancies in Japan are zoledronic acid and pamidronate. Pamidronate is approved for malignancy-induced hypercalcemia and osteolytic bone metastases from breast cancer, whereas zoledronic acid is approved for malignancy-induced hypercalcemia and bone lesions resulting from multiple myeloma or bone metastases from solid tumors. In Europe, intravenous ibandronate is approved for the inhibition of bone metastasis-related events. One known adverse event associated with bisphosphonates is nephrotoxicity; the present draft examines the necessity of dose reduction in accordance with renal function.

Commentary

High-dose (90-360 mg/month) pamidronate has been reported to induce glomerulosclerosis and acute tubular necrosis, as well as to accelerate acute renal failure and nephrotic syndrome [178]. A subsequent examination of dosage and administration time found that nephrotoxicity was mild when pamidronate 90 mg was administered over the course of ≥ 3 hours, while a phase III trial found no significant pamidronate-induced nephrotoxicity compared to placebo. Based on these results, the American Society of Clinical Oncology (ASCO) guidelines were revised to specify that when pamidronate 90 mg is administered over the course of ≥ 2 hours, reduction of the pamidronate dose is unnecessary even when Ccr is 30-60 mL/min; the guidelines also specify that when Ccr is < 30 mL/min, further prolongation of pamidronate administration time (4-6 hours) or pamidronate dose reduction is recommended [179, 180].

Several phase III trials of zoledronic acid for breast cancer with osteolytic lesions, multiple myeloma, lung cancer, and other solid tumors began with a protocol of 4 mg or 8 mg administered over the course of 5 minutes; however, the group that received 8 mg over 5 minutes demonstrated a high incidence of nephrotoxicity, thereby necessitating a two-stage protocol amendment. First, the administration duration was extended from 5 minutes to 15 minutes; second, the 8 mg dose was reduced to 4 mg. These amendments reduced the incidence of nephrotoxicity induced by zoledronic acid to a rate equal to that induced by placebo or by pamidronate (the control group) [181–185]. In 2005, Novartis Pharmaceuticals filed a package insert revision with the FDA stating that for patients with decreased renal function (Ccr 30-60 mL/min), the dosage of zoledronic acid was to be reduced to achieve the same AUC as that for patients with a Ccr of 75 mL/min (specifically, doses of 3.5 mg, 3.3 mg, and 3.0 mg for patients with a Ccr of 50-60 mL/min, 40-49 mL/min, and 30-39 mL/min, respectively). The revised ASCO guidelines specify the following: the recommended dose and duration for zoledronic acid is 4 mg over ≥ 15 minutes; when Ccr is 30-60 mL/min, the dose should be reduced in accordance with the recommendation in the package insert; and when Ccr is < 30 mL/min, zoledronic acid should not be administered. In a retrospective analysis of 220 patients, Shah et al. reported that when zoledronic acid doses were adjusted as recommended in the package insert, patients with decreased renal function demonstrated the same incidence of acute renal failure (as an adverse event associated with zoledronic acid) as patients with normal renal function [186].

Ibandronate, for which an intravenous formulation is approved in Europe, is used to inhibit associated skeletal-related events associated with bone metastasis (in Japan, ibandronate is approved only in oral form for osteoporosis). Ibandronate is considered to be associated with the lowest incidence of nephrotoxicity of all intravenous bisphosphonates [187] However, the package insert recommends that when Ccr is > 50 mL/min, infusion time should be extended from 15 minutes to 1 hour; and when Ccr is < 30 mL/min, the dose should be reduced from 6 mg to 2 mg.

Anti-RANKL antibodies were developed to treat bone metastasis; in a phase III trial, anti-RANKL antibodies were significantly superior to zoledronic acid in inhibiting skeletal-related events. Renal impairment did not occur; thus, dose adjustments in accordance with renal function are considered unnecessary [188]. However, patients with Ccr < 30 mL/min and ESRD patients requiring dialysis were excluded from the trial; therefore, it is necessary to consider the potential onset of serious hypocalcemia and to assess the suitability of anti-RANKL antibodies carefully for patients with severe renal impairment.

(4) Maintenance dialysis patients

**CQ14: Is dialysis therapy recommended for drug removal following cisplatin administration in maintenance dialysis patients?**

Recommendation grade: Weakly advised against (suggestion)

Recommendation

The majority of cisplatin binds to tissue and proteins and remains in the body even if dialysis is performed, with the resultant potential for a post-dialysis rebound. Therefore, dialysis therapy for drug removal is not recommended following cisplatin administration for maintenance dialysis patients, regardless of timing.

Summary

The majority of cisplatin binds to tissue and proteins and remains in the body even if dialysis is performed, with the resultant potential for a post-dialysis rebound. Therefore, dialysis therapy for drug removal is not recommended following cisplatin administration for maintenance dialysis patients, regardless of timing. However, this is only an expert opinion based on case reports; further clinical study is necessary to close the evidence-to-practice gap.

Background and Objectives

Due to concerns regarding accumulated toxicity following cisplatin administration, ESRD patients sometimes undergo dialysis for drug removal. In the present draft, we assess the efficacy of dialysis therapy for drug removal following cisplatin administration.

Commentary

Cisplatin rapidly binds to plasma proteins upon entering the blood, thereby undergoing conversion from unbound cisplatin (free Pt) to protein-bound cisplatin (≈total Pt). One side effect of cisplatin is nephrotoxicity; dialysis patients, whose renal function has already been eliminated, may instead face problems such as myelotoxicity and peripheral neuropathy.

Aside from case reports, there are very few systematic studies of the pharmacokinetics of cisplatin in dialysis patients. In their investigation of the pharmacokinetics of cisplatin in five patients who developed gastric cancer during maintenance dialysis, Miyakawa et al. reported the following results. When cisplatin was administered concurrently with the initiation of dialysis, the concentration of free Pt in blood rapidly decreased and was below measurable levels following dialyzer use; the concentration of total Pt fluctuated relatively sharply in the early stage and subsequently decreased gradually. When dialysis was initiated 1 hour after cisplatin administration, changes in concentrations of free Pt and total Pt in blood were considered to be the same as when cisplatin and dialysis were initiated simultaneously. However, the study does not specify which patients began cisplatin and dialysis immediately and which patients began dialysis 1 hour after administration of cisplatin [189]. In the same year as the above study, Miyakawa et al. reported on the pharmacokinetics of cisplatin in 2 gastric cancer patients undergoing maintenance dialysis; however, it is unclear whether these 2 patients were also included in the other study [190].

In all case reports, aside from a report by Inozume et al. stating that, “In order to maximize the effect of the key drug cisplatin, we elected to perform dialysis the day after cisplatin” [191], dialysis was initiated 30 minutes to 1 hour following the administration of cisplatin. Without allowing a certain interval following cisplatin administration, free Pt will be eliminated from the blood by dialysis before it can bind to plasma proteins, thus greatly reducing the antitumor effect. Patients with normal renal function who receive cisplatin demonstrate a biphasic pattern in which the blood concentration of cisplatin increases sharply in the early stage (α-phase), then gradually decreases (β-phase). This pattern is also observed in patients with chronic renal failure; the α-phase is considered to be result of the entry of cisplatin into tissue [192]. The β-phase is the result of excretion of cisplatin from the kidneys; in patients with renal failure, this reduction in the blood concentration of cisplatin is either diminished or completely absent. This biphasic pattern is also observed in reports in which dialysis is initiated 30 minutes to 1 hour following cisplatin administration.

Cisplatin administered *in vivo* binds to proteins in plasma and tissue in a short time, after which it is not dialyzed; therefore, in 3.5 to 4 hours of dialysis, only approximately 10% of cisplatin is removed [193, 194]. Most cisplatin removed from the body is free Pt; the majority of cisplatin binds to tissue and proteins, thus remaining in the body even when dialysis is performed. A post-dialysis rebound results in a renewed increase in free Pt in blood [191, 195–200]. In addition, as the volume of accumulated cisplatin increases, the rate of cisplatin removed by dialysis further decreases [198, 201].

The above-cited studies demonstrate that although most free Pt can be removed by dialysis, the majority of cisplatin binds to tissue and proteins and thus remains in the body even when dialysis is performed, thereby potentially resulting in a post-dialysis rebound and a consequent renewed increase in cisplatin concentration. Therefore, the answer to the present CQ can be considered to be, “Even when dialysis is performed following cisplatin administration, not only is the cisplatin removal rate roughly a mere 10%, a rebound phenomenon occurs; therefore, regardless of timing (whether immediately after cisplatin or 30 minutes to 1 hour after cisplatin), dialysis is not recommended for the removal of cisplatin”. However, this is an only an expert opinion based on case reports; further clinical study is necessary to close the evidence-to-practice gap. When cisplatin is administered to dialysis patients, a 50-75% dose reduction is recommended [202, 203]. When dialysis is performed following cisplatin administration, caution is necessary regarding cisplatin accumulation.

(5) Particular comorbidities

**CQ15: Is rasburicase recommended for preventing tumor lysis syndrome?**

Recommendation grade: Strongly recommended

Recommendation

Rasburicase is recommended for preventing tumor lysis syndrome.

Summary

The suitability of rasburicase for preventing tumor lysis syndrome (TLS) has been described according to risk in the Japanese Society of Medical Oncology’s Tumor Lysis Syndrome Practice Guidance [204]; rasburicase has also been reported to reduce the need for hemodialysis. Rasburicase reduces uric acid levels, prevents nephropathy, and is effective in preventing TLS.

Background and Objectives

Rasburicase is a recombinant version of urate oxidase that rapidly metabolizes uric acid to allantoin. Compared to uric acid, allantoin is much more soluble in urine; this metabolism thus rapidly reduces uric acid levels in blood. Administration of rasburicase requires caution regarding the following three points: 1) rasburicase is an enzyme preparation and may thus trigger hypersensitivity reactions; 2) antibody formation has been reported, thereby rendering repeated administration is not recommended; 3) rasburicase is contraindicated in patients with glucose-6-phosphate dehydrogenase deficiency. The present draft examines whether rasburicase is recommended for the prevention of TLS.

Commentary

The suitability of rasburicase for preventing TLS has been described according to risk in the Japanese Society of Medical Oncology’s Tumor Lysis Syndrome Practice Guidance [204]; for high-risk and moderate-risk patients, in cases in which uric acid levels continually increase despite the use of allopurinol and febuxostat, or in cases in which hyperuricemia is observed at diagnosis, rasburicase should be administered or at least considered [204]. The TLS preventive effect of rasburicase has been demonstrated in a phase III study that randomly assigned subjects at high risk for TLS to rasburicase only (0.20 mg/kg/day, days 1-5), rasburicase plus allopurinol (rasburicase 0.20 mg/kg/day, days 1-3; allopurinol 300 mg/day days 3-5), or allopurinol only (300 mg/day, days 1-5). In comparison to the allopurinol only group, the rasburicase only group demonstrated a significantly lower incidence of laboratory TLS^1^ [205]. In a number of other studies conducted in children, rasburicase significantly reduced uric acid levels compared to allopurinol [206, 207]. Regarding the nephropathy preventive effect of rasburicase, a systematic review of multiple clinical trials conducted in leukemia and lymphoma patients found that hemodialysis was performed for 0-2.8% of patients who used rasburicase, versus 15.9-25.0% of patients who did not use rasburicase; thus, the use of rasburicase tended to reduce the need for hemodialysis [208]. Rasburicase has also been shown to reduce uric acid levels in patients at high risk for TLS in a number of randomized comparative trials [209, 210]. The above-cited studies demonstrate that rasburicase reduces uric acid levels, prevents nephropathy, and is effective for preventing TLS.

**CQ16: Is plasmapheresis recommended for anticancer drug-induced thrombotic microangiopathy?**

Recommendation grade: Weakly advised against (suggestion)

Recommendation

Due to the absence of definitive evidence, plasmapheresis is currently not recommended for anticancer drug-induced thrombotic microangiopathy (TMA). Although plasmapheresis has been observed to inhibit the progression of TMA-induced renal impairment in a handful of isolated cases, the efficacy of plasmapheresis for this purpose has not been properly assessed and therefore is currently not recommended.

Summary

Plasmapheresis is currently not recommended for anticancer drug-induced TMA due to the dearth of reliable evidence on its efficacy for this purpose. Although there are several case series and cross-sectional studies regarding mitomycin C, these studies have been no assessments of therapy with plasmapheresis alone. Because, plasmapheresis is often performed following hemodialysis or is combined with drug therapy based on anti-platelet drugs and steroids. On the other hand, regarding TMA-induced renal impairment, several reports have only stated that plasmapheresis inhibited further worsening of renal function. In addition, plasmapheresis is often combined with hemodialysis. Thus, the usefulness of plasmapheresis against drug-induced TMA has not truly been assessed.

Background and Objectives

Thrombotic microangiopathy is a disorder that presents with thrombocytopenia, microangiopathic hemolytic anemia, and organ dysfunction. Classical TMAs include thrombotic thrombocytopenic purpura (TTP) and Shiga toxin-induced hemolytic-uremic syndrome (HUS), in both of which activity of a disintegrin-like and metalloproteinase with thrombospondin type 1 motifs 13 (ADAMTS13) is reduced. However, there is a great deal that remains unknown regarding the pathology of TMA, while the pathology of TMA is diverse; therefore, in 2013, all TMAs other than TTP and HUS were defined as atypical hemolytic-uremic syndrome (aHUS), for which diagnostic criteria were created [212]. While TTP can present with both congenital and acquired ADAMTS13 deficiency, most cases involve acquired deficiency, in which anti-ADAMTS13 autoantibodies are involved. Therefore, plasmapheresis is the first-line treatment for acquired TTP. The objectives of plasmapheresis are ADAMTS13 replenishment; removal of anti-ADAMTS13 antibodies; and removal of unusually large von Willebrand factor multimers (UL-vWFM), which are multimers composed of a hemostatic factor called von Willebrand factor. For HUS, plasmapheresis has not been established as effective and is used primarily as supportive therapy. Plasmapheresis is also used for aHUS induced by complement system abnormalities; however, due to the diverse etiology of aHUS, the efficacy of plasmapheresis for this purpose has not been established. Drug-induced TMA includes TTP caused by production of immunological autoantibodies against ADAMTS13 associated with ticlopidine and other antiplatelet drugs; for this form of TTP, plasmapheresis is effective. On the other hand, calcineurin inhibitors such as cyclosporine and tacrolimus are not associated with ADAMTS13 deficiency; these drugs considered to cause aHUS, which primarily induces vascular endothelial injury without decreasing ADAMTS13 activity, for which plasmapheresis is often ineffective. Many cases of drug-induced TMA are considered to present with a pathology resembling that of aHUS; however, the detail mechanism of drug-induced TMA remains poorly understood. Anticancer drugs that induce TMA include mitomycin C, cisplatin, bleomycin, gemcitabine, pentostatin, and sunitinib [213].

Although plasmapheresis is combined with antiplatelet drugs and steroids to treat TMA, there is no established treatment. The present draft examines the efficacy of plasmapheresis for anticancer drug-induced TMA.

Commentary

In regard to the efficacy of plasmapheresis for mitomycin C-induced TMA, a case series of 4 patients [214] reported the results of antiplatelet drugs + plasmapheresis (3-4 L) administered 5-7 times over 1-2 weeks. Two patients demonstrated rapid improvement in platelet count, red blood cell count, and other hematologic parameters, as well as a tendency toward recovery of renal function within 6 weeks. Another patient continued to demonstrate decreased renal function following plasmapheresis; however, over the following ≥ 4 months, renal function gradually improved. The last patient, despite demonstrating an increase in platelet count following plasmapheresis, did not show improvement in renal function and subsequently died. No relationship was demonstrated in these patients between overall mitomycin C dose and TMA onset; thus, no definitive conclusion was reached about the usefulness of plasmapheresis. In some cases, plasmapheresis is combined with antiplatelet drugs (e.g., dipyridamole and sulfinpyrazone) or hemodialysis (conditions unknown); therefore, the effects of plasmapheresis monotherapy are difficult to assess.

In regard to cancer-associated HUS, a cross-sectional study of patients in a national registry with hematocrit ≤ 25%, platelet count < 10 × 10^4^ μL, and serum Cr ≥ 1.6 mg/dL (accounting for 99% of patients receiving mitomycin C and 68% of patients receiving 5-FU) [215] found that of the 37 patients who underwent plasmapheresis, 11 patients (30%) responded to treatment, while 26 patients (70%) either did not respond to treatment or worsened. In a case series of 12 patients who developed TMA following mitomycin C-containing chemotherapy regimens [216], all patients demonstrated renal failure at the time of diagnosis; although 2 of these patients demonstrated low serum Cr values of 1.8 mg/dL and 2.7 mg/dL, the remaining 10 patients demonstrated serum Cr values of 3.4-9.6 mg/dL. Six of these patients underwent 2 L plasmapheresis 3 times over the course of 1-2 weeks while also receiving antiplatelet drugs or steroids. However, only 1 of these patients responded to plasmapheresis; this patient was also receiving steroids, azathioprine, and dipyridamole.

In a cross-sectional study of breast cancer patients, plasmapheresis was performed 2-49 times (median 46 times) to treat TMA which had developed following high-dose chemotherapy with cyclophosphamide, cisplatin, and carmustine + autologous bone marrow stem cell transplantation [217]. High-dose chemotherapy was performed for 581 patients, 15 of whom (2.6%) developed TMA; 4 of these patients survived. Of the 15 patients who developed TMA, 12 underwent steroid therapy + plasmapheresis. Survival following TMA diagnosis was 2-76 days (median 41 days); 3 of the 4 survivors had undergone plasmapheresis a mean 50 times.

In a case series of 9 patients who developed TMA among a total of 2,586 patients receiving gemcitabine [218], the median time to development of TMA in the 9 patients was 8 months (3-18 months) following a total gemcitabine dose of 19.2 g/m^2^ (9-56 g/m^2^). Six of the patients survived, while 3 died. Of these 9 patients, 5 underwent plasmapheresis. Among these 5 patients, 2 died, while the other 3 developed chronic renal failure; of these latter 3 patients, 2 required dialysis. Of the 3 patients whose renal function recovered, none underwent plasmapheresis; however, the report does not explain the details of this recovery.

## Footnotes

1 Based on Cairo-Bishop diagnostic criteria [211].
